# Immune profiling of human vestibular schwannoma secretions identifies TNF-α and TWEAK as cytokines with synergistic potential to impair hearing

**DOI:** 10.1186/s12974-025-03364-z

**Published:** 2025-02-08

**Authors:** Sasa Vasilijic, Richard Seist, Zhenzhen Yin, Lei Xu, Konstantina M. Stankovic

**Affiliations:** 1 Department of Otolaryngology– Head and Neck Surgery, Stanford University School of Medicine, 801 Welch Rd, Palo Alto, Stanford, CA 94304 USA; 2 Edwin L. Steele Laboratories, Department of Radiation Oncology, Massachusetts General Hospital, Harvard Medical School, Boston, MA USA; 3 Department of Neurosurgery, Stanford University School of Medicine, Stanford, CA USA; 4 Wu Tsai Neurosciences Institute, Stanford University, Stanford, CA USA

**Keywords:** Vestibular schwannoma, Great auricular nerve, Sensorineural hearing loss, Cytokines, Chemokines, Tumor-conditioned media, Perilymph, Blood

## Abstract

**Background:**

Vestibular schwannoma (VS) is an intracranial tumor arising from the Schwann cells of the vestibular nerve and is an important cause of sensorineural hearing loss (SNHL) in humans. The mechanisms underlying this SNHL are incompletely understood and currently, there are no drugs FDA approved specifically for VS. This knowledge gap significantly limits the development of effective treatments aimed at preventing, stabilizing, or reversing VS-induced SNHL.

**Methods:**

To identify effector molecules involved in VS-induced SNHL, we analyzed 47 immune-related factors secreted by tumor tissue in over 50 patients with sporadic VS and studied their correlation with preoperative hearing ability and tumor size. The most promising effector molecules were validated in vivo in an anatomically accurate mouse model of VS, and in vitro with mouse fibroblasts (L929) and auditory cell lines representing pro-sensory precursors of hair cells (UB-OC1) and auditory neuroblasts (US-VOT-N33).

**Results:**

We demonstrated that VS-induced SNHL was linked to increased secretion of TNF-α, IL-2R, CD163, eotaxin, and HGF, while larger tumor size was associated with higher levels of TNF-α, TNF-R2, IL-1α, IFN-α, MIP-1β, and IL-21 secretion. We identified heterogeneity among VS tumors in their capacity to secrete TNF-α. Tumors with high levels of TNF-α secretion released cytokines and chemokines that significantly correlated with poor hearing (TWEAK and eotaxin) or better hearing (LIF, GRO-α, MIP-1α, MIP-3α, and IL-1α). Among these, TWEAK was notably abundant, with levels exceeding those in normal nerve tissue, elevated in patients with non-serviceable hearing and strongly linked to poor hearing in patients with TNF-α high-secreting tumors. In vivo, we demonstrated that VS-secreted factors reach the inner ear, with elevated TNF-α and TWEAK in the perilymph and blood of tumor-bearing mice with impaired hearing. In vitro, TWEAK amplified TNF-α -mediated cytotoxicity in TNF-α sensitive cells (L929) and auditory cell lines (UB-OC1 and US-VOT-N33) at tumor-secreted concentrations.

**Conclusion:**

This study provides compelling evidence that VS-secreted TNF-α and TWEAK act synergistically to drive tumor-induced SNHL. Targeting the TNF-α/TWEAK axis presents a promising new avenue for preventing VS-induced SNHL.

**Supplementary Information:**

The online version contains supplementary material available at 10.1186/s12974-025-03364-z.

## Introduction

Vestibular schwannoma (VS), an intracranial tumor primarily arising from Schwann cells of the vestibular nerve, is the most common tumor of the cerebellopontine angle [1]. While histologically benign, VS is considered malignant by location because it can cause multiple cranial nerve deficits and, rarely, even death due to brainstem compression [2]. A critical factor prompting VS development is a mutation of the neurofibromin 2 (*NF2*) gene, which impairs the production or function of the tumor suppressor protein Merlin [3, 4]. *NF2* mutations are typically confined to tumor cells, leading to unilateral and sporadic VS in over 90% of cases [5], with an incidence of 1:10,000 [6]. In contrast, rare germline mutations are associated with bilateral VS and neurofibromatosis type 2 (NF2)-related schwannomatosis [7], the latter which has an estimated incidence of 1:33,000 live births [8]. Over the past four decades, the incidence of VS has risen dramatically, increasing from 3 cases per million per year to 34 cases per million per year [9]. This growth is largely attributed to advancements in diagnostic technologies and increased tumor detection in older individuals. During the same period, the average size of tumors at diagnosis decreased from 26 mm to 7 mm, while the average age at diagnosis rose from 49 to 60 years [9].

The primary clinical manifestation of VS is sensorineural hearing loss (SNHL), present in 95% of patients, which is frequently accompanied by tinnitus and balance disturbances [10–13]. Currently, there are no pharmacological or biological treatments approved by the United States Food and Drug Administration for VS or the associated SNHL [14]. Current management approaches for VS include observation of non-growing tumors or surgical microdissection and stereotactic radiation therapy of growing tumors, all of which carry risks, including worsening hearing loss [15–18].

The mechanisms underlying VS-induced SNHL are multifactorial, encompassing tumor-induced mechanical compression of the auditory nerve [19], ischemia in the inner ear caused by disruption of the cochlear blood supply [20, 21], and secretion of ototoxic factors by tumor tissue [22, 23]. Although some studies reported that both larger initial tumor size and higher tumor volumetric growth rates are correlated with an increased risk of hearing loss [24], our previous findings have highlighted the lack of a consistent correlation between VS tumor size, position, and the severity of patients’ SNHL at the time of diagnosis [25]. Furthermore, we previously identified tumor-secreted factors such as tumor necrosis factor-alpha (TNF-α) and IL-6 as candidate ototoxic molecules that could be associated with hearing loss in VS patients [23, 26]. Additionally, we previously conducted immune profiling of blood plasma from a large cohort of VS patients, which identified significantly elevated levels of certain cytokines, chemokines, and growth factors, some of which may be biomarkers for VS, VS-induced SNHL, and tumor growth [27]. These observations, together with increasing evidence of an inflammatory environment within VS [28–32], have led us to hypothesize that immune-related factors are key components of the VS tissue secretome and that their profiling and linking to hearing outcomes could help identify effector molecules involved in VS-induced hearing loss.

To test this hypothesis, we conducted comprehensive immune profiling of VS-secreted factors in tissue-conditioned media, analyzing over 50 tumor samples from patients with sporadic VS, using an array of 67 cytokines, chemokines, and growth factors (Additional file 1: Table S1). The results of the present analyses identified tumor-secreted factors significantly associated with hearing loss and tumor size in VS patients. Furthermore, through a combination of in vitro studies involving auditory cell lines, and an in vivo anatomically accurate mouse model of VS, we identified and validated TNF-α and TWEAK as effector molecules that synergistically contribute to VS-induced hearing loss These insights pave the way for future research and potential therapeutic interventions targeting the TNF-α/TWEAK axis to prevent or rescue VS-induced hearing loss.

## Results

### Patient characteristics

A total of 98 patients with sporadic VS were included in the study (Additional file 1: Fig. S1). Among them, 42 had serviceable hearing (SH), defined as word recognition (WR) score > 50% and pure tone average (PTA) < 50 dB in the ipsilateral ear, 53 had non-serviceable hearing (NSH), and 35 controls without VS (Additional file 1: Fig. S1, Fig. S2). The mean ages of the overall VS patients and controls were 53 and 58 years, respectively, and the proportion of females was similar (both 54%). VS-SH patients were significantly younger than the VS-NSH (mean age: 48 versus 55 years, *P* = 0.026) and controls (mean age: 48 versus 58 years, *P* = 0.005) (Additional file 1: Fig. S2A). Compared to VS-SH patients, VS-NSH patients had significantly larger mean tumor volume (9.2 cm^3^ versus 4.5 cm^3^; *P* = 0.008), poorer ipsilateral PTA (71.5 dB versus 31.2 dB; *P* < 0.0001) and contralateral PTA (21.1 dB versus 14.2 dB; *P* = 0.039), and lower WR scores (20.5% versus 85.0%; *P* < 0.0001) (Additional file 1: Fig. S2A, Fig. S3). The finding of worse contralateral hearing among VS-NSH patients is consistent with a previous report, which attributed it to tumor-secreted ototoxic factors that may percolate through the cerebrospinal fluid or the blood to reach the contralateral ear and affect its hearing [33].

### Immunoprofiling of human VS tumor-secreted factors

#### Tumor versus normal nerve tissue

**Fig. 1:**
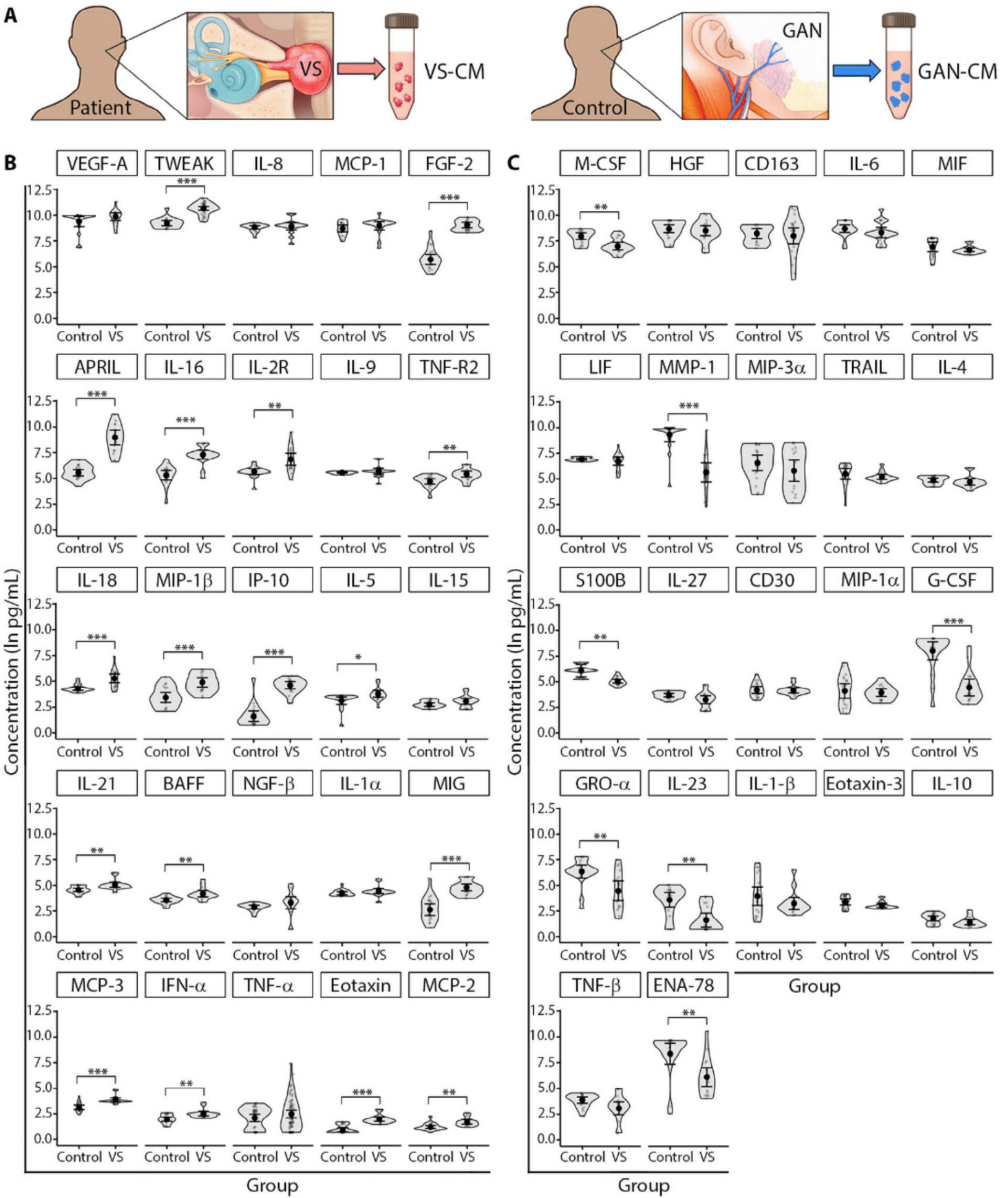
Immunoprofiling of secreted immune-related factors from VS and normal GAN tissue. (**A**) Study design for immunoprofiling of secreted factors. The levels of 24 of 47 detected immune-related factors significantly differed between CM collected from VS tumor and normal GAN tissue (control). (**B**) The concentrations of 17 factors were significantly elevated in VS-CM versus GAN-CM. (**C**) Seven factors were significantly elevated in GAN-CM versus VS-CM. Generalized linear mixed effects regression - GLS model was used to compare secreted factor levels. Each data point represents a VS tumor sample or normal GAN sample. The full names of the profiled factors are listed in Additional file 1: Table S1. *Padj < 0.05, **Padj < 0.01, ***Padj < 0.001. VS, vestibular schwannoma; GAN, great auricular nerve; CM, conditioned media; VS-CM, vestibular schwannoma- conditioned media; GAN-CM, great auricular nerve- conditioned media; *P*adj, adjusted *P* value corrected for multiple comparisons. A schematic illustration of GAN is modified after a figure in the Skull Base Surgery Atlas (Jackler & Gralapp, Stanford Medicine, https://skullbasesurgeryatlas.stanford.edu/)

Fourthy-seven of the 67 profiled factors were detectable in the vestibular schwannoma tissue-conditioned media (VS-CM) of more than 60% of analyzed VS samples (Additional file 1: Table S2). In both VS-CM and CM from healthy great auricular nerve (GAN) tissue (GAN-CM), TWEAK, VEGF-A, MCP-1, IL-8, CD163, and HGF were among the top ten factors with the highest detected levels. Specifically, the highest mean values were recorded for TWEAK, IL-8, and VEGF-A in VS-CM, and for MMP-1, VEGF-A, and TWEAK in GAN-CM (Fig. 1).

The levels of 24 soluble factors significantly differed between the tumor (VS-CM) and healthy nerve tissue (GAN-CM) (Fig. 1). In VS-CM samples, 17 factors were significantly elevated compared to controls (TWEAK, FGF-2, APRIL, IL-16, IL-2R, TNF-R2, IL-18, MIP-1β, IP-10, IL-5, IL-21, BAFF, MIG, MCP-3, IFN-α, eotaxin, and MCP-2) (Fig. 1B). The most elevated were FGF-2, IP-10, APRIL, TWEAK, IL-16, MIG, and eotaxin (all *Padj* < 0.001), whose VS/GAN ratio ranged from 30 (FGF-2) to 2.5 (eotaxin) times higher (Additional file 1: Fig. S4A). In GAN-CM samples, 7 secreted factors were significantly elevated compared to VS-CM samples (M-CSF, MMP-1, S100B, G-CSF, GRO-α, IL-23, and ENA-78) (Fig. 1C). The most elevated were G-CSF, GRO-α, ENA-78, and MMP-1 (all *Padj* < 0.001), having GAN/VS ratios ranging from 258 (G-CSF) to 53 (MMP-1) times higher (Additional file 1: Fig. S4B).

#### Tumors from patients with serviceable versus non-serviceable hearing

**Fig. 2:**
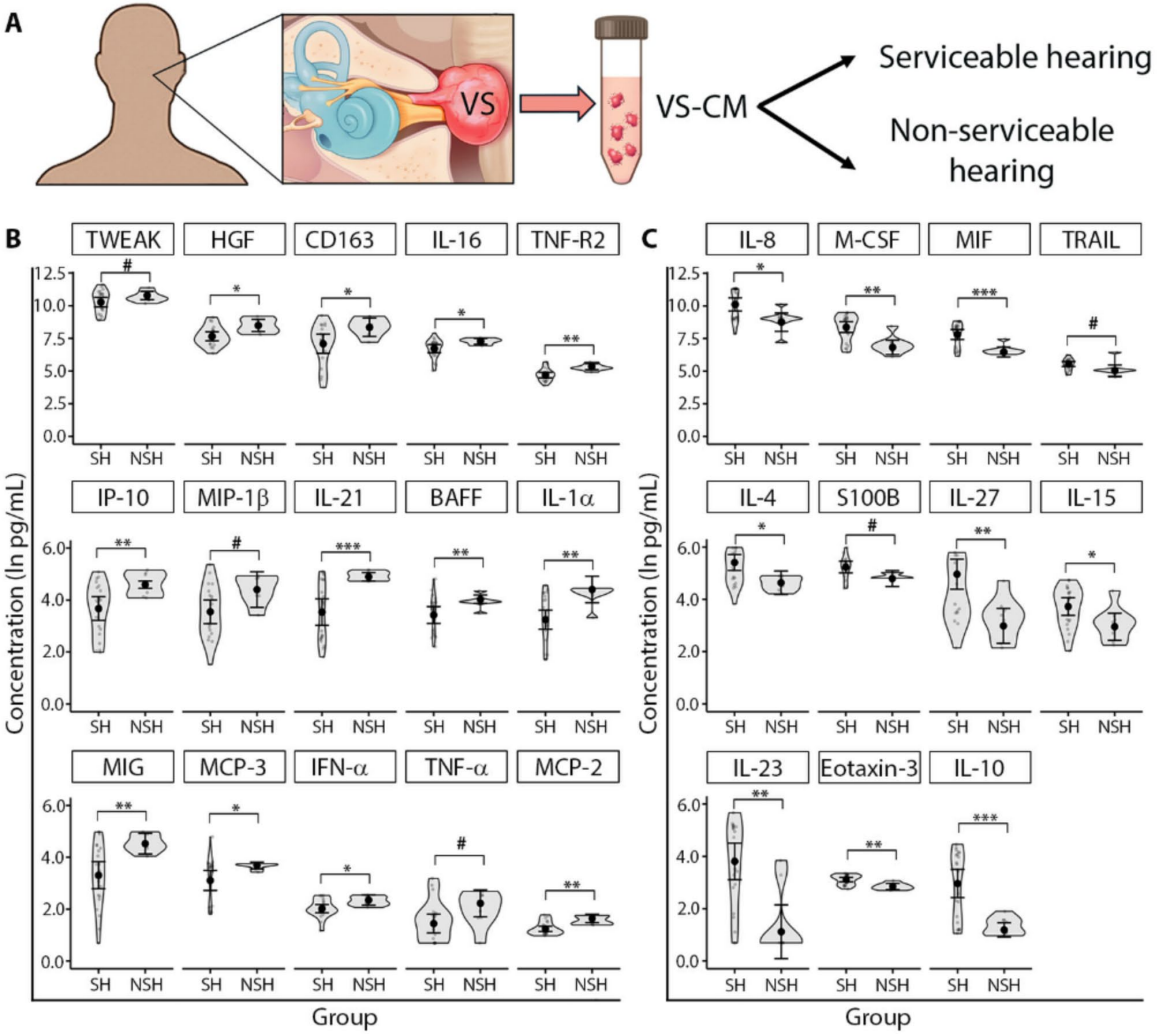
Secretory capacity of VS tissue from patients with serviceable and non-serviceable hearing. (**A**) Study design for immunoprofiling of secreted factors. (**B**) Fifteen soluble factors were highly produced by VS tissue from patients with NSH. Among these, the levels of TWEAK, IL-16, TNF-R2, IP-10, MIP-1β, IL-21, BAFF, MIG, MCP-3, IFN-α, and MCP-2 were significantly higher in VS-CM compared to GAN-CM (as listed in Fig. 1B). (**C**) Thirteen soluble factors were highly secreted by VS tissue from patients with SH, with M-CSF, S100B, and IL-23 notably decreased in VS-CM relative to GAN-CM (as listed in Fig. 1C). SH was defined as AAO-HNS Class A and B hearing (PTA ≤ 50 dB and WR score ≥ 50%). NSH was defined as AAO-HNS Class C and D hearing (PTA > 50 dB or WR score < 50%). Generalized linear mixed effects regression- GLS model was used for the comparisons. Each data point represents a VS tumor tissue sample. The full names of secreted factors are listed in Additional file 1: Table S1. *Padj < 0.05, **Padj < 0.01, ***Padj < 0.001. ^#^ significant at *P* < 0.05 prior to adjustment. AAO-HNS, American Academy of Otolaryngology–Head and Neck Surgery; VS, vestibular schwannoma; VS-CM, vestibular schwannoma- conditioned media; SH, serviceable hearing; NSH, non-serviceable hearing; PTA, pure tone average; WR, word recognition; *P*adj, adjusted *P* value corrected for multiple comparisons

The levels of 26 tumor-secreted factors significantly differed between VS samples from patients with SH and NSH (Fig. 2). Compared to patients with SH, VS samples isolated from patients with NSH secreted significantly higher levels of TWEAK, HGF, CD163, IL-16, TNF-R2, IP-10, MIP-1β, IL-21, BAFF, IL-1α, MIG, MCP-3, IFN-α, TNF-α, and MCP-2 (Fig. 2B). Among these factors, the levels of CD163, HGF, IP-10, TNF-α, TWEAK, and IL-16 were all ≥ 1.2 times higher in VS-SH compared to VS-NSH samples (Additional file 1: Fig. S5A). In contrast, samples isolated from patients with SH secreted significantly higher levels of IL-8, M-CSF, MIF, TRAIL, IL-4, S100B, IL-27, IL-15, IL-23, eotaxin-3 and IL-10 compared to VS-NSH samples (Fig. 2C). The greatest elevations were observed for IL-27 (2 times higher) followed by IL-23 and IL-15 (each 1.5 times higher); the levels of IL-4, IL-10, S100B, IL-8, and M-CSF were all ≥ 1.2 times higher (Additional file 1: Fig. S5B).

### Correlation between blood levels of secreted factors and tumor secretion capacity in vitro

**Fig. 3:**
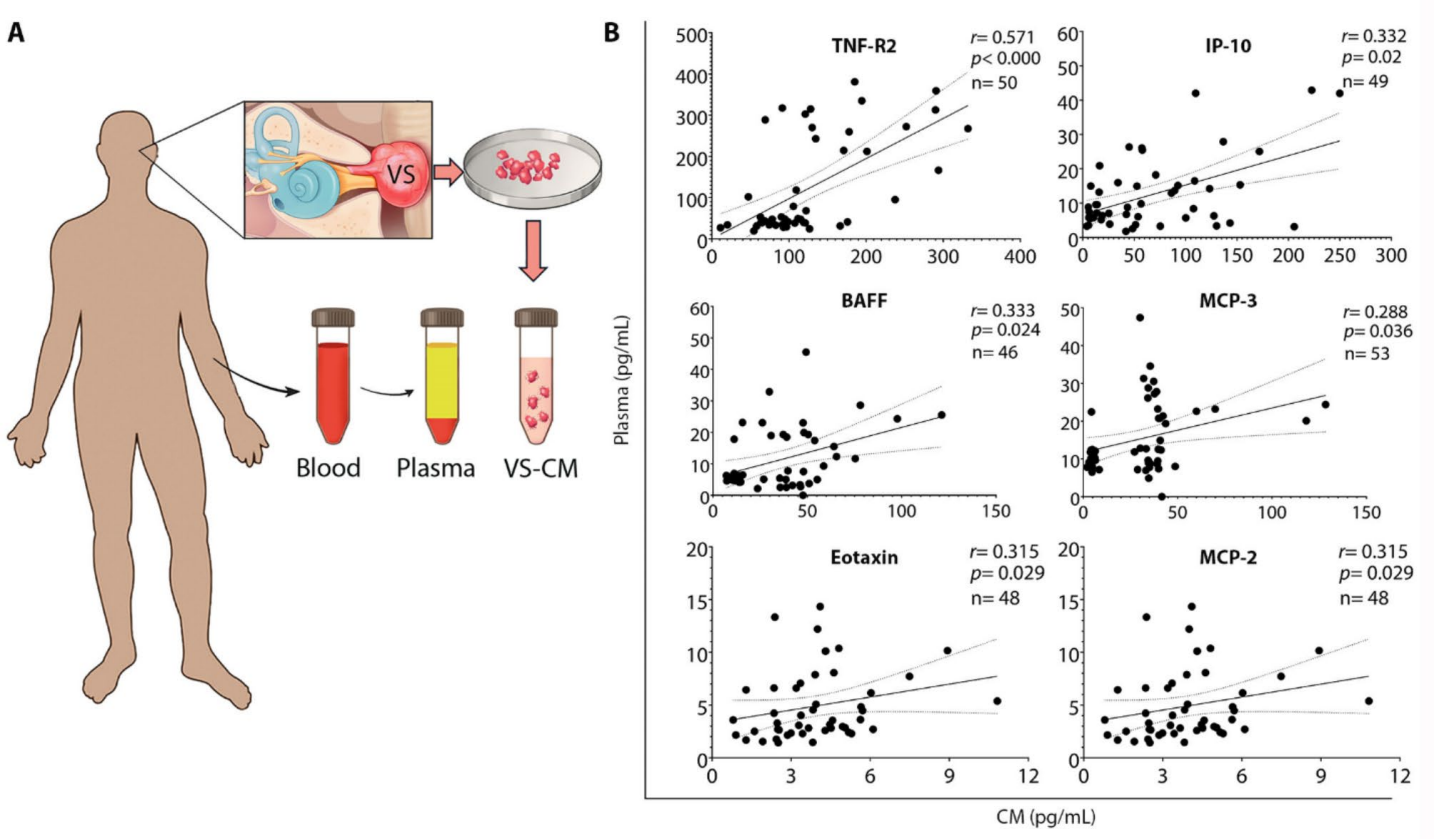
Systemic levels of secreted factors correlate with tumor-secreted capacity in vitro. (**A**) Study design to analyze the correlation between the levels of secreted factors in plasma and tumor-conditioned media. (**B**) Six out of the 17 secreted factors significantly elevated in vitro positively correlate with their corresponding plasma levels, as analyzed using Spearman’s correlation coefficient ‘r’. The solid line depicts the regression curve, while the dotted lines indicate the 95% confidence intervals of the best-fit line. Each black circle represents a unique VS patient. VS, vestibular schwannoma; CM, conditioned media; VS-CM, vestibular schwannoma- conditioned media

Given that the systemic secretion of soluble factors by tumor cells and the tumor microenvironment may contribute to systemic inflammation in tumor-related diseases [34], we investigated the correlation between the levels of soluble factors in plasma and VS-CM. Plasma levels of six tumor-secreted factors (TNF-R2, IP-10, BAFF, MCP-3, eotaxin, and MCP-2) positively correlated with their levels in VS-CM (Fig. 3B). These findings indicate that the plasma levels of these six factors accurately reflect the tumor’s secretion capacity.

### Association of the tumor-secreted factor levels with pre-operative hearing and tumor size in VS patients

**Fig. 4:**
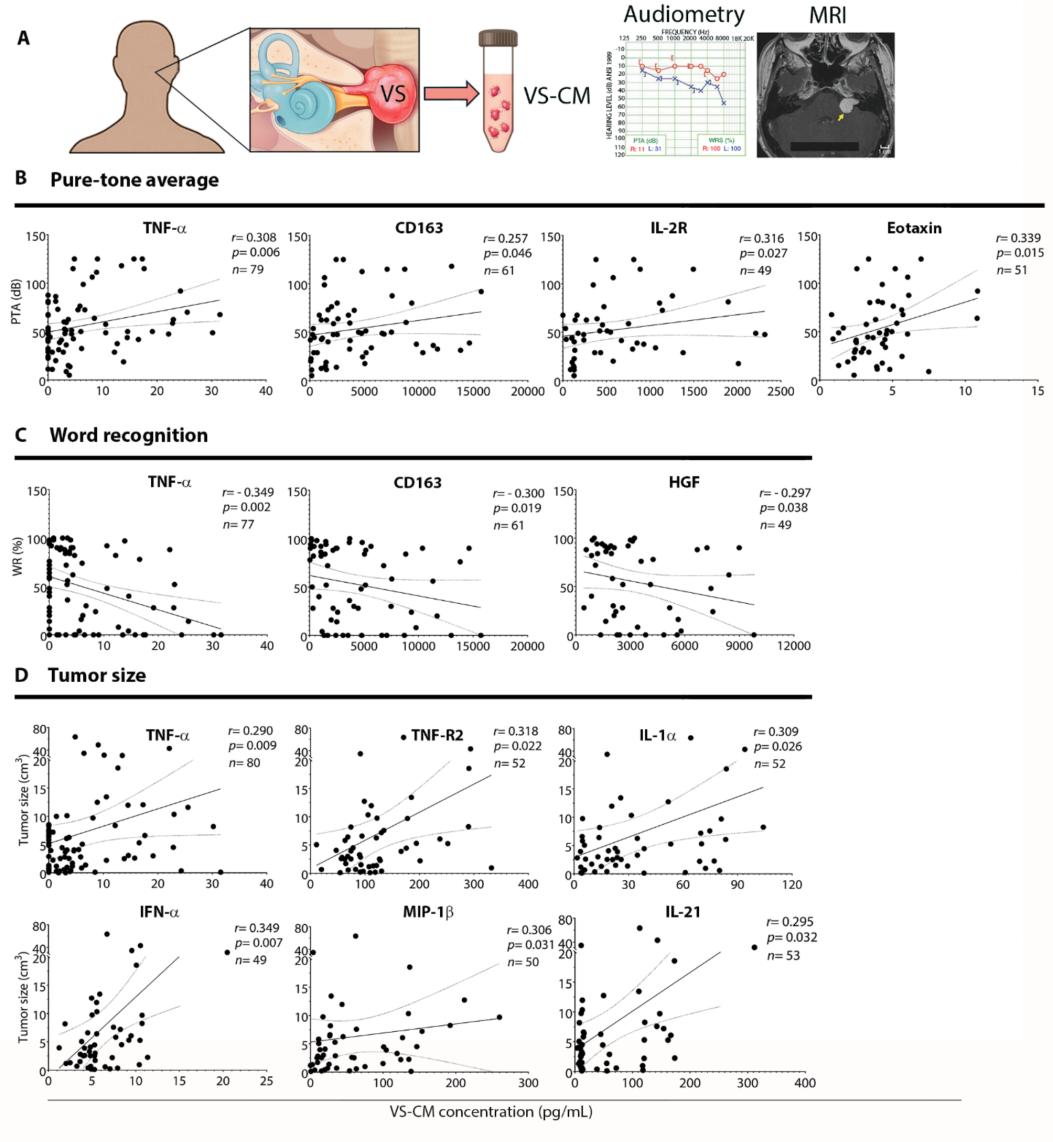
VS-secreted factors associate with the severity of preoperative hearing loss and tumor size. (**A**) Study design to analyze the correlation between the levels of secreted factors and clinical characteristics of VS patients. The levels of tumor-secreted factors, preoperative hearing parameters (pure tone average [**B**] and word recognition score [**C**]), and tumor size [**D**] were analyzed for correlation using Spearman’s correlation coefficient. Among the cytokines, TNF-α was uniquely associated with worse hearing ability as well as larger tumor size. Additionally, elevated levels of IL-2R, CD163, eotaxin, and HGF correlated with hearing impairment (direct relationship with PTA values and/or inverse relationship with WR scores). The levels of TNF-R2, IL-1α, IFN-α, MIP-1β, and IL-21 showed only a positive correlation with tumor size in VS patients. ‘r’ represents Spearman’s rank correlation coefficient. The solid line depicts the regression curve, while the dotted lines indicate the 95% confidence intervals of the best-fit line. Each black circle represents a unique VS patient. VS, vestibular schwannoma; VS-CM, vestibular schwannoma-conditioned media; MRI, magnetic resonance imaging; PTA, pure tone average; dB, decibel; WR, word recognition score. The schematic (A) incorporates an audiogram and MRI from our previously published work (https://pubmed.ncbi.nlm.nih.gov/33604573/)

The relationships between levels of tumor-secreted factors and pre-operative PTA, WR, and tumor size were assessed among VS patients using Spearman correlation analysis and regression analysis. Out of 47 factors detected in VS-CM, the levels of only 5 factors (TNF-α, IL-2R, CD163, eotaxin, and HGF) were associated with VS patients’ hearing ability (Fig. 4, B and C). Increased PTA values and decreased WR scores in VS patients were associated with elevated secretions of TNF-α (PTA: *r* = 0.308, *P* = 0.006; WR: *r*=-0.349, *P* = 0.002) and CD163 (PTA: *r* = 0.257, *P =* 0.046; WR: *r*=-0.300, *P =* 0.019). Increased levels of IL-2R (*r* = 0.316, *P =* 0.027) and eotaxin (*r* = 0.339, *P =* 0.015) were associated only with PTA, and elevated HGF only with lower WR scores (*r*=-0.297, *P =* 0.038) (Fig. 4, B and C).

Larger tumor size was associated with elevated levels of TNF-α (*r* = 0.290, *P =* 0.009), TNF-R2 (*r* = 0.318, *P =* 0.022), IL-1α (*r* = 0.309, *P =* 0.026), IFN-α (*r* = 0.349, *P =* 0.007), MIP-1β (*r* = 0.306, *P =* 0.031), and IL-21 (*r* = 0.295, *P =* 0.032) in VS-CM (Fig. 4D). Furthermore, TNF-α levels were significantly elevated (*P* < 0.001) in CM from tumor tissue of VS patients who underwent subtotal resection (STR) rather than gross total resection (GTR) (Additional file 1: Fig. S6A). However, tumor tissue from the patients who underwent GTR secreted higher levels of FGF-2 (*P* < 0.001), as well as ENA-78, MCP-3, and IL-21 (all, *P* < 0.05) (Additional file 1: Fig. S6A). In addition, tumor volume was higher in VS patients who underwent STR compared to GTR (12.4 versus 5.0 cm^3^, *P* = 0.011) (Additional file 1: Fig. S6B).

### Correlation of the tumor-secreted factor levels with pre-operative hearing and tumor size in patients whose tumors have high or low TNF-α secretion capacity

The VS tumor samples showed high variability in their secreted levels of TNF-α (coefficient of variation [CV]: 444.3%, range: 0–1,681 pg/mL). Therefore, all VS-CM samples were categorized into two groups representing high or low TNF-α secretion capacity, according to whether they exceeded the average level of TNF-α released into CM by healthy GAN tissue (11.66 pg/mL). Specifically, the TNF-α High group and the TNF-α Low group consisted of VS-CM with TNF-α levels above 11.66 pg/mL (average level 126.4 ± 320.3 pg/mL) and below 11.66 pg/ mL (average level 2.91 ± 3.1 pg/mL), respectively (Additional file 1: Fig. S7). The two groups were separately analyzed for released immune-related factors and their association with pre-operative hearing and tumor size.

**Fig. 5:**
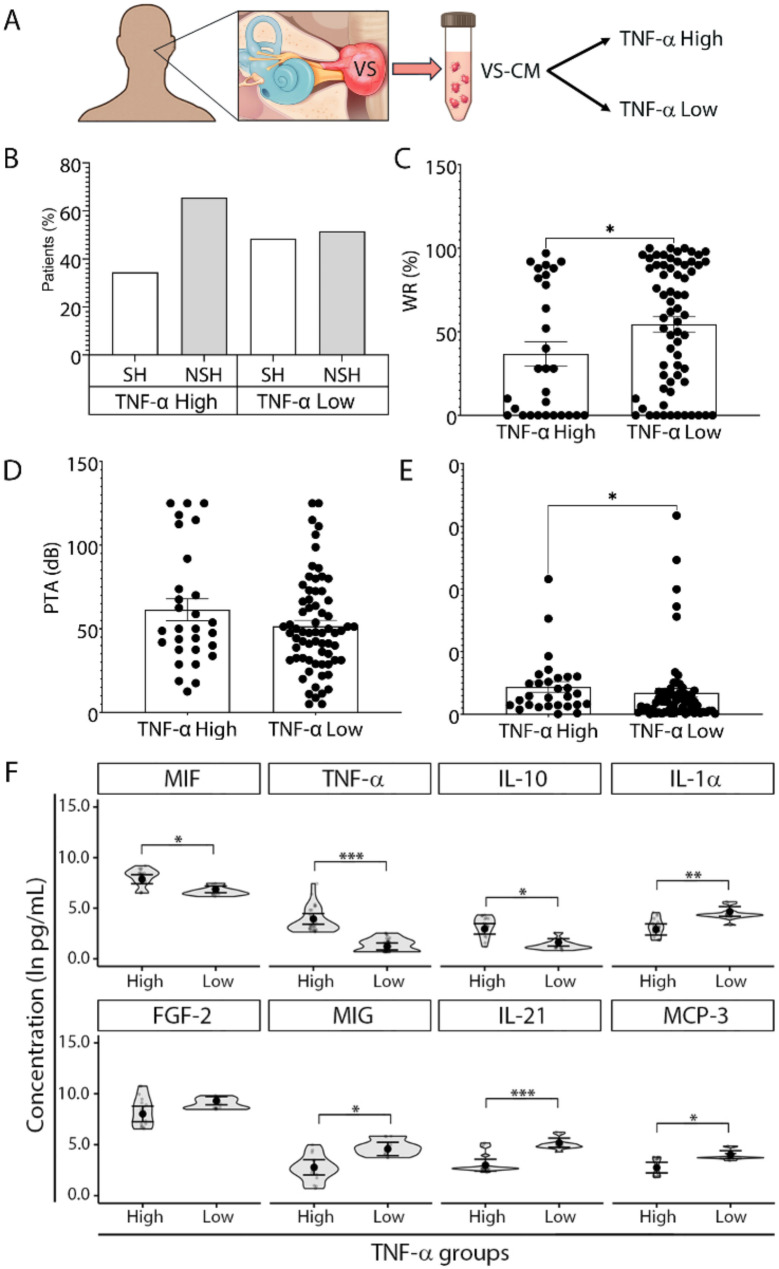
Clinical characteristics and immune factors in VS patients with high versus low TNF-α tumor secretion. (**A**) Study design to compare preoperative clinical characteristics and secreted factors between VS patients with TNF-α high and low tumor-secretion capacity. (**B**) The distribution of patients with serviceable hearing (SH) and non-serviceable hearing (NSH) in TNF-α High (*n* = 29) and TNF- α Low groups (*n* = 69) highlights the higher prevalence of NSH in the TNF-α High group. VS patients were categorized into TNF-α High and TNF-α Low groups based on whether their tumor-secreted TNF-α levels were above or below the average TNF-α levels secreted by normal nerve tissue (Additional file 1: Fig. S7). (**C**, **D**) Comparison of hearing outcomes: patients in the TNF-α High secretion group exhibited worse hearing with lower WR scores than patients in the TNF-α Low secretion group, despite similar PTA values. (**E**) Tumor volume was, on average, larger in the TNF-α High group as compared with the TNF-α Low group. (**F**) Differential expression of immune-related factors: eight out of 47 significantly altered factors between tumor and normal tissue demonstrated significant differences between TNF-α high (*n* = 17–21) and TNF-α low groups (*n* = 37–46). C, D, E, Mann-Whitney two-tailed test. F, Generalized linear mixed effects regression- GLS model. Each data point represents a unique VS patient. The full names of secreted factors are listed in Additional file 1: Table S1. *P < 0.05, **P < 0.01, ***P < 0.001. VS, vestibular schwannoma; VS-CM, vestibular schwannoma-conditioned media; PTA, pure tone average; dB, decibel; WR, word recognition

Comparison of hearing characteristics between VS patients with TNF-α high and TNF-α low secretion capacity revealed that 65.5% of VS patients whose tumors secreted high levels of TNF-α had NSH (Fig. 5B). Correspondingly, WR scores (but not PTA values) were significantly lower in VS patients with TNF-α high secretion capacity (TNF-α High versus TNF-α Low: 36.79 *±* 38.57%, 95% CI: 22.12–51.47; versus 54.43 *±* 36.92%, 95% CI: 45.35–63.51, *P* < 0.05), suggesting the association of poor hearing with increased production of TNF-α by tumor tissue (Fig. 5C and D). Furthermore, bigger tumor volumes were observed in VS patients with TNF-α high secretion capacity (TNF-α High versus TNF-α Low: 8.75 *±* 9.19 cm^3^ versus 6.87 *±* 11.44 cm^3^, *p* < 0.05) (Fig. 5E).

To investigate whether the secretory capacity for other cytokines differed between TNF-α High and TNF-α Low tumor types, we compared the secreted levels while controlling for age, sex, and tumor size using a generalized least squares (GLS) model. Eight differently secreted factors between these two groups were identified. In addition to TNF-α (*Padj* < 0.001), tumor tissue from the TNF-α High group secreted significantly higher levels of MIF and IL-10 (both *Padj* < 0.05). The TNF-α Low group showed increased secretion of IL-1α (*Padj* < 0.01), IL-21 (*Padj* < 0.001), MIG, and MCP-3 (both *Padj* < 0.05), while FGF-2 approached the criterion for statistical significance (*Padj* = 0.053) (Fig. 5F).

**Fig. 6:**
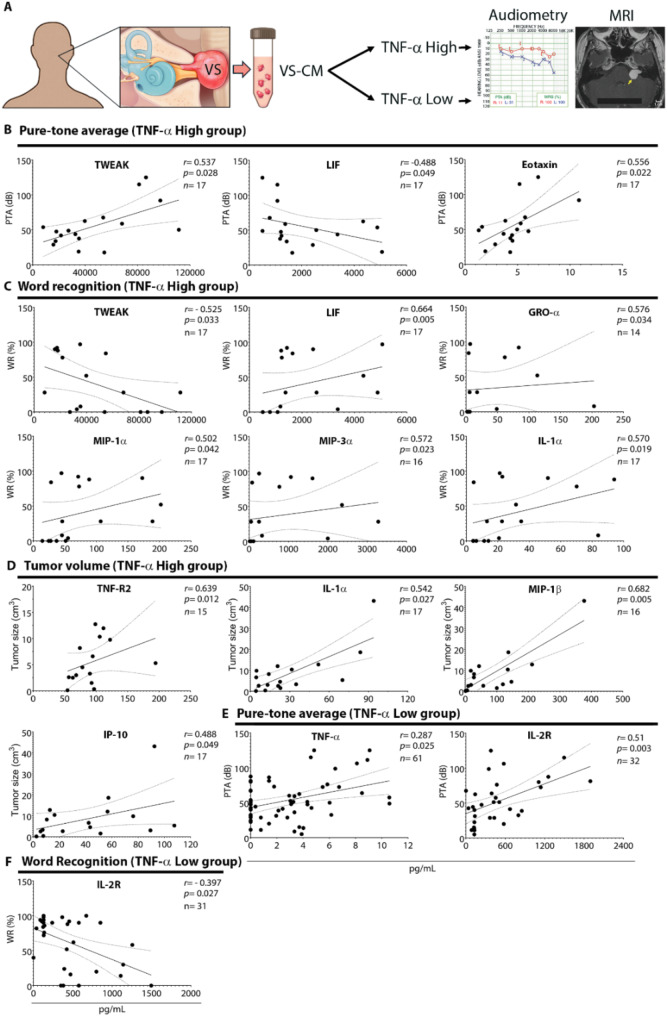
Different factors secreted by TNF-α High and Low tumors associate with hearing and tumor size. (**A**) Study design to analyze the correlation between the levels of secreted factors and preoperative clinical characteristics of VS patients with TNF-α high and low tumor-secretion capacity. The levels of tumor-secreted factors, PTA, WR scores, and tumor size, were correlated using Spearman’s correlation coefficient, ‘r’. Factors secreted by TNF-α High tumor type correlated with preoperative clinical characteristics to a greater extent than TNF-α Low tumor type. The levels of multiple factors secreted by TNF-α High tumors were significantly correlated with PTA (three correlations, **B**), WR scores (six correlations, **C**), and tumor size (four correlations, **D**). In contrast, two factors secreted by TNF-α Low tumors significantly correlated with PTA (**E**) or WR (**F**). The solid line depicts the regression curve, while the dotted lines indicate the 95% confidence intervals of the best-fit line. Each black circle represents a unique VS patient. VS, vestibular schwannoma; VS-CM, vestibular schwannoma-conditioned media; MRI, magnetic resonance imaging; PTA, pure tone average; dB, decibel; WR, word recognition score. The schematic (A) incorporates an audiogram and MRI from our previously published work (https://pubmed.ncbi.nlm.nih.gov/33604573/)

Correlation analysis of secreted factor levels, hearing parameters, and tumor size in the TNF-α High and Low groups revealed associations not apparent prior to grouping. In the TNF-α High group, TWEAK levels in VS-CM were strongly associated with the severity of hearing loss (PTA: *r =* 0.537, *P* = 0.028; WR: *r=*-0.525, *P* = 0.033) (Fig. 6B and C). Eotaxin was again associated with poor hearing, yet with a stronger correlation than in the unseparated cohort (PTA: *r =* 0.556, *P* = 0.022, versus *r =* 0.292, *P* = 0.042) (Figs. 4B, and 6B). Interestingly, a subset of secreted factors was found to be associated with improved hearing in the TNF-α High group, unlike the overall group of VS patients: LIF (PTA: *r=*-0.488, *P* = 0.049; WR: *r =* 0.664, *P* = 0.005), GRO-α (WR: *r =* 0.576, *P* = 0.034), MIP-1α (WR: *r =* 0.502, *P* = 0.042), MIP-3α (WR: *r =* 0.572, *p* = 0.023), and IL-1α (WR: *r =* 0.570, *P* = 0.019) (Fig. 6C).

Correlation of the tumor size values with the secreted levels of cytokines in the TNF-α High group identified a moderate association of tumor size with IP-10 (*r =* 0.488, *P* = 0.049). The previously observed association of TNF-R2, IL-1α, and MIP-1β levels with tumor size was confirmed but with a much stronger correlation strength than in the unseparated group (TNF-R2: *r =* 0.639, *P* = 0.012, versus *r =* 0.318, *P* = 0.022; IL-1α: *r =* 0.542, *P* = 0.027, versus *r =* 0.309, *P* = 0.026; MIP-1β: *r =* 0.682, *P* = 0.005, versus *r =* 0.306, *P* = 0.031) (Figs. 4D and 6D).

In the TNF-α Low group, the association with worsening hearing was confirmed only for TNF-α (PTA: *r =* 0.287, *P* = 0.025) and IL-2R (PTA: *r =* 0.510, *P* = 0.003; WR: *r*=-0.397, *P* = 0.027) (Fig. 6E, F).

### TNF-α and TWEAK are elevated in the perilymph and blood of mice with cerebellopontine angle schwannomas

To investigate whether factors secreted by VS can reach the inner ear, we used an anatomically accurate mouse model of VS. Specifically, *Nf2*-deficient mouse Schwann cells transfected with the *Gaussia princeps luciferase* (Gluc) reporter gene were allografted intracranially into the cerebellopontine angle (CPA) region [35]. This in vivo model replicates the progressive SNHL induced by schwannoma and enables precise longitudinal measurements of tumor volume, detecting as few as 100 tumor cells through blood measurements of Gluc bioluminescence secreted by the schwannoma cells [36].

**Fig. 7:**
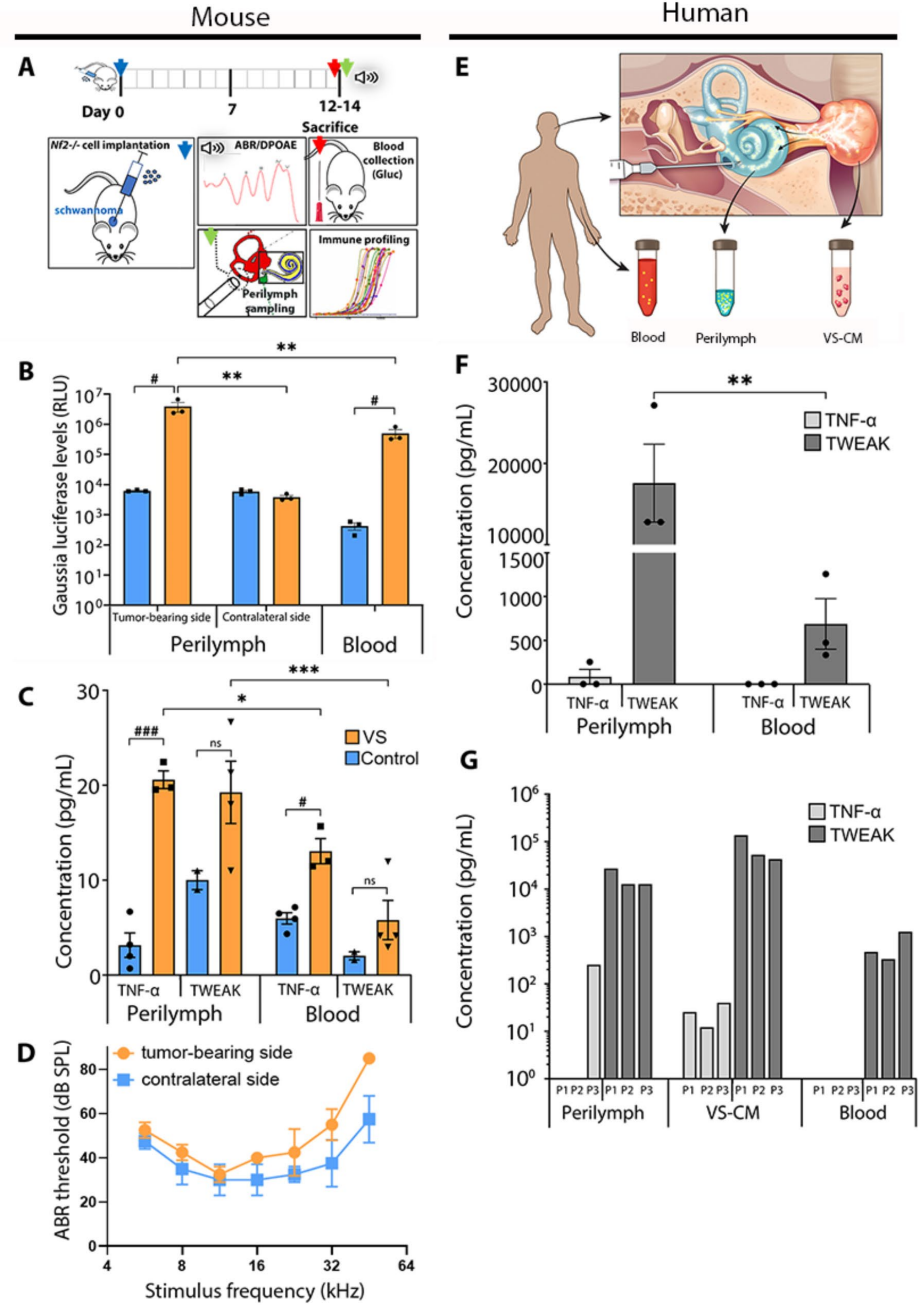
VS-secreted factors reach the inner ear, elevating TNF-α and TWEAK in mouse and human perilymph. (**A**) Experimental paradigm for testing the presence of tumor-secreted factors in the inner ear of tumor-bearing animals. Gaussia luciferase-secreting *Nf2*^*−/−*^ mouse Schwann cells were stereotactically injected into the CPA region of mice (*n* = 4). Mice that underwent unilateral sham surgery (saline injection) were used as controls (*n* = 4). (**B**) Gaussia luciferase secreted by tumor cells was detected markedly above the baseline luminescence values in the perilymph ipsilateral but not contralateral to the tumor, and in the blood of the tumor-bearing but not control mice. (**C**) Tumor-bearing mice had elevated levels of TNF-α and TWEAK in perilymph and blood compared to controls. (**D**) Tumor-bearing mice had elevated ABR thresholds indicating impaired hearing on the tumor-bearing side compared to the contralateral side. (**E**) Study design to compare the levels of secreted factors in blood, perilymph, and conditioned media of VS patients. (**F**) Levels of TNF-α and TWEAK were notably higher in the perilymph than the blood of VS patients. (**G**) Comparison of TNF-α and TWEAK levels in the perilymph, tumor-conditioned media, and blood of three VS patients indicated that patients with the highest in vitro tumor secretion capacity for TNF-α (patient p3) and TWEAK (patient p1) had the highest levels of these cytokines in their perilymph. A, B, ^#^*P* < 0.05, ^###^*P* < 0.001, Unpaired two-tailed *t*-test; *P < 0.05, **P < 0.01, ***P < 0.001, Two-way ANOVA followed by Šídák’s multiple comparisons test. B, C, Each dot represents a different mouse; data shown as mean, error bars, s.e.m. F, Two-way ANOVA followed by Fisher’s LSD test. Each dot represents a unique VS patient; data are shown as mean, error bars, s.e.m. ABR, auditory brain stem response; CPA, cerebellopontine angle; SPL, sound pressure level; RLU, relative light unit; VS, vestibular schwannoma

Compared to mice receiving sham surgery, the CPA tumor-bearing (TB) mice had substantially higher levels of Gluc bioluminescence in their blood 12–14 days after grafting of *Nf2*-deficient Schwann cells. Perilymph (inner ear fluid) collected from the ears ipsilateral to the tumor, but not from the contralateral ears, had Gluc levels substantially above the baseline levels detected in the perilymph of the sham-operated control animals (*p* < 0.01). This finding indicates that tumor-secreted Gluc can reach the ipsilateral inner ear (Fig. 7B). As expected, significantly higher levels of Gluc bioluminescence were also detected in the blood of the TB animals compared to controls. However, the blood Gluc levels were substantially lower than in the perilymph from the TB side (*P* < 0.05), indicating that the concentration of tumor secretions is the highest in the inner ear ipsilateral to the tumor.

Given the significant correlation observed between hearing impairment and elevated levels of tumor-secreted TNF-α (Fig. 4B and C) and TWEAK (Fig. 6B and C) in VS patients, we further analyzed the levels of these cytokines in the blood and perilymph of TB and control mice. The levels of both TNF-α and TWEAK were increased more than two times in the blood of TB mice compared to controls (TNF-α: 13.05 ± 2.29 pg/mL versus 5.97 ± 1.20 pg/ mL, *P* < 0.05; TWEAK: 5.8 ± 4.14 pg/mL versus 2.02 ± 0.60 pg/ mL, *P* = 0.067) (Fig. 7C). In the perilymph, TNF-α and TWEAK levels were higher on the TB side than the sham-operated mice (TNF-α: 20.59 ± 1.62 pg/mL versus 3.14 ± 2.57 pg/ mL, *P* < 0.001; TWEAK: 19.25 ± 6.56 pg/mL versus 10.0 ± 1.41 pg/ mL, *P* = 0.063) (Fig. 7C). Furthermore, the levels of TNF-α and TWEAK were higher in the perilymph than the blood, and TWEAK was elevated to a greater extent than TNF-α. Specifically, TWEAK levels were 3.3 higher in the perilymph than blood of TB mice (19.25 ± 6.56 pg/mL versus 5.80 ± 4.14 pg/mL, *P* < 0.0001, Two-Way ANOVA), while TNF-α levels were 1.6 times higher in the perilymph than blood (20.59 ± 1.62 pg/mL versus 13.05 ± 2.29 pg/mL, *P* < 0.016, Two-Way ANOVA) (Fig. 7C).

Concordant with the finding of greater hearing impairment on the TB side of patients with sporadic VS, the TB mice had increased auditory brainstem response (ABR) thresholds on the TB side compared to the contralateral tumor-free side (Fig. 7D).

Next, we measured TNF-α and TWEAK levels in the perilymph and blood of patients with sporadic VS undergoing translabyrinthine craniotomy for tumor resection and observed a similar trend as in the mouse model of VS. The levels of TWEAK were significantly higher in perilymph than in blood samples (*P* < 0.01, Two-Way ANOVA) (Fig. 7F). Furthermore, a comparison of individual perilymph levels from three VS patients with in vitro tumor-secreted levels showed that perilymph levels of TWEAK and TNF-α are proportional to the secretory capacity of the VS tissue, suggesting their origin from the VS tumor (Fig. 7G).

### TWEAK enhances TNF-α cytotoxicity at tumor-secreted levels

**Fig. 8:**
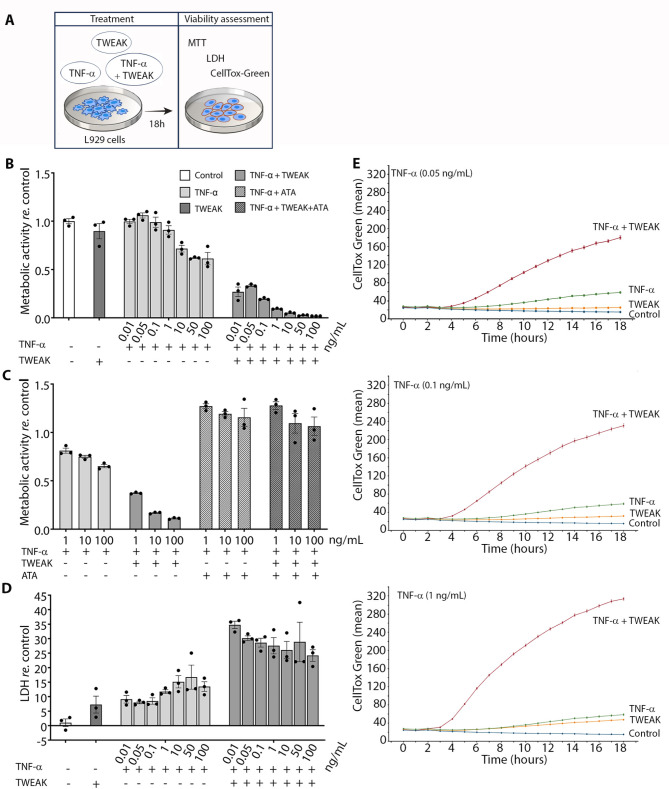
TWEAK strongly amplifies TNF-α cytotoxic activity within the range of concentration secreted by VS tumors. (**A**) Experimental paradigm for testing the contribution of TWEAK in TNF-α mediated cytotoxicity. Co-treatment of L929 cells with 50 ng/mL TWEAK and 0.01–100 ng/mL TNF-α dramatically reduced cell viability compared to the cells treated separately with TWEAK or TNF-α. (**B**) Cytokine co-treatment results in significantly reduced metabolic activity in L929 cells. (**C**) TWEAK and TNF-α co-treatment in the presence of ATA, an Fn14 signaling pathway inhibitor, abrogates the cytotoxic effect of the co-treatment. (**D**) Cells co-treated with TWEAK and TNF-α released significantly higher levels of LDH than cells treated with either factor alone, indicating an increased rate of cell death in the co-treated cultures. (**E**) Cells co-treated with TWEAK and TNF-α exhibited increased CellTox Green Dye staining, confirming compromised membrane integrity and indicating necroptosis as the underlying form of cell death associated with TWEAK and TNF-α cytotoxicity. The cytotoxic effect of TWEAK and TNF-α was manifested 4–5 h after the co-treatment and reached a maximum 18 h later. Metabolic activity and LDH levels are presented relative to untreated control L929 cells. CellTox Green Dye staining is expressed as a mean of fluorescence intensity (mean). Each data point represents technical replicates; data are shown as mean ± s.e.m. MTT, [3-(4,5-dimethylthiazol-2-yl)-2,5-diphenyltetrazolium bromide]; LDH, lactate dehydrogenase; ATA, aurintricarboxylic acid

Given that elevated levels of TNF-α and TWEAK are associated with the greater severity of human hearing loss (Figs. 4B, C and 6B, C), and that these factors are increased in the perilymph on the tumor-bearing side (Fig. 7C), it is plausible to propose their cooperation in mediating VS-induced hearing loss. To determine whether TWEAK can enhance TNF-α cytotoxicity, the murine fibrosarcoma L929 cell line was used as a well-established cellular model to study TNF-α cytotoxicity and necroptosis [37]. The cells were treated with TNF-α and TWEAK at the concentration range spanning their tumor-secreted levels detected in VS-CM. Compared to untreated L929 cells, cells treated with TNF-α alone exhibited significantly reduced metabolic activity at high concentrations, starting from 10 ng/mL TNF-α (Fig. 8B). Conversely, treatment with TWEAK alone at 50 ng/mL, which is close to the average secreted levels in VS-CM (46.39 ± 32.71 ng/mL), did not significantly reduce cells’ metabolic activity (Fig. 8B). However, a co-treatment of cells with TNF-α and TWEAK led to a dramatic reduction of metabolic activity even at the lowest tested concentrations of TNF-α (0.01 ng/mL, *P* < 0.0001), suggesting that TWEAK strongly amplifies activity of TNF-α. Moreover, TWEAK and TNF-α co-treatment of L929 cells in the presence of aurintricarboxylic acid (ATA), an Fn14 signaling pathway inhibitor [38], abrogated the cytotoxic effect of the co-treatment, highlighting the crucial role of TWEAK/Fn14 signaling in TNF-α mediated cytotoxicity (Fig. 8C).

Because both TNF-α and TWEAK have been reported to promote necroptosis [39, 40], we measured the release of lactate dehydrogenase (LDH) and the permeability of the cell membrane after treatment. Decreased metabolic activity of L929 cells co-treated with TWEAK and TNF-α was followed by significantly increased release of LDH in the cell culture media (Fig. 8D) and cell death (as measured by CellTox Green staining) (Fig. 8E) compared to the cells treated with either of the two cytokines alone.

### Synergistic interaction underpins the combined cytotoxicity of TWEAK and TNF-α

As we observed a significantly amplified cytotoxicity of TNF-α in the presence of TWEAK, we next quantified the degree of TNF-α and TWEAK interaction in the L929 cell line by calculating the combination index (CI) and dose reduction index (DRI) using the Chou-Talalay method (Table 1, Additional file 1: Fig. S8). A 7 × 7 dose-response matrix was applied, resulting in 49 treatment conditions (Additional file 1: Fig. S9A). The effect of TNF-α and TWEAK interaction on the viability of L929 cells was assessed using an MTT assay and expressed as fractional inhibition (Fa), representing inhibition of metabolic activity from 0 (no inhibition) to 1 (total inhibition). CI and DRI were calculated for the constant concentration ratio (iso-concentrations) and the variable ratio of TNF-α and TWEAK (different concentrations of TNF-α and permanent concentration of TWEAK).

**Table 1:** Synergy parameters of TWEAK and TNF-α applied at constant and variable concentration ratios

Ratio	TWEAK + TNF-α (ng/mL)	Fractional inhibition (Fa)	CI	Description	DRI (TWEAK)	DRI (TNF-α)
Constan**t**	0.05 + 0.05	0.01	11,026	Very strong antagonism	0.066	9.08E-05
	0.1 + 0.1	0.025	508.851	Very strong antagonism	0.7142	0.002
	1 + 1	0.092	22.311	Very strong antagonism	5.961	0.045
	10 + 10	0.509	0.022	Very strong synergism	1126.61	47.77
	50 + 50	0.95	1.30E-06	Very strong synergism	2.86E + 06	1.05E + 06
	100 + 100	0.99	1.65E-06	Very strong synergism	1.20E + 08	1.21E + 08
Variable	50 + 0.01	0.264	0.139	Strong synergism	7.25	701.11
	50 + 0.05	0.323	0.055	Very strong synergism	18.28	2,184.27
	50 + 0.1	0.491	0.006	Very strong synergism	177.94	35,762.60
	50 + 1	0.57	0.002	Very strong synergism	496.28	126,574
	50 + 10	0.906	3.74E-06	Very strong synergism	264,407	2.98E + 08
	50 + 50	0.95	6.99E-07	Very strong synergism	1.43E + 06	2.63E + 09
	50 + 100	0.97	2.04E-07	Very strong synergism	4.89E + 06	1.30E + 10

TWEAK and TNF-α were applied together at the constant (iso concentrations) and variable ratios (different concentrations of TNF-α). The Combination Index (CI) and Dose-Reduction Index (DRI) were calculated using the Chou-Talalay method through the CompuSyn software. According to the CI values, synergy is indicated by CI < 1, an additive effect by CI = 1, and antagonism by CI > 1. The DRI values represent the potential for dose reduction, where DRI > 1 suggests favorable dose reduction, DRI = 1 indicates no dose reduction, and DRI < 1 reflects an unfavorable dose reduction. ‘Fa’ denotes the fraction affected, representing the fractional inhibition of metabolic activity

Treatment of cells at low iso-concentrations of TWEAK and TNF-α (both at 0.05, 0.1, and 1 ng/mL) caused a negligible inhibition of metabolic activity, resulting in CI > 10, which indicates a very strong antagonistic interaction. By contrast, co-treatment of cells with higher iso-concentrations (both at 10, 50, and 100 ng/mL) dramatically inhibited metabolic activity, ranging from Fa 0.5 to 0.95, and CI < 0.1, demonstrating a very strong synergistic interaction between TWEAK and TNF-α (Table 1, Additional file 1: Fig. S8, A and B). A very strong synergistic interaction (CI < 0.1) was observed when the cells were treated with permanent concentrations of TWEAK (50 ng/mL) and variable concentrations of TNF-α spanning the range of VS-secreted levels (0.05, 0.1, and 1 ng/mL) (Table 1, Additional file 1: Fig. S8, C and D). The synergistic effect of TWEAK and TNF-α was followed by the favorable dose reduction (DRI > 1) at all tested concentrations except for TWEAK and TNF-α applied at iso-concentrations at 1 and 10 ng/mL (DRI < 1) (Table 1).

Applying 50 ng/mL TWEAK and the lowest TNF-α concentration (0.01 ng /mL) resulted in 26% inhibition of L929 cells’ metabolic activity, indicating a strong synergistic interaction (CI = 0.14) and favorable dose reduction for both cytokines (DRI > 1). This effect was achieved by 7.2 fold and 701.1 fold lower concentrations of TWEAK and TNF-α, respectively, than if they were applied separately. The degree of TWEAK-TNF-α synergism and dose reduction was progressively increased with higher concentrations of TNF-α, reaching CI of 0.006 and DRI of 496.3 and 126,574 for TWEAK and TNF-α, respectively, when TNF-α was applied at 1 ng/ mL, representing the upper levels detected in TNF-α High tumors (Table 1, Additional file 1: Fig. S7). Synergistic interaction was additionally confirmed by calculating synergy scores using four commonly used synergy scoring reference models, including Highest Single Agent (HSA), Loewe Additivity, Bliss Independence, and Zero Interaction Potency (ZIP) [41]. Synergy scores calculated by all four reference models were higher than 10 (Loewe: 14.3, HSA: 13.5; ZIP: 11, and Bliss: 10.6) (Additional file 1: Fig. S9B), confirming true synergy.

**Fig. 9:**
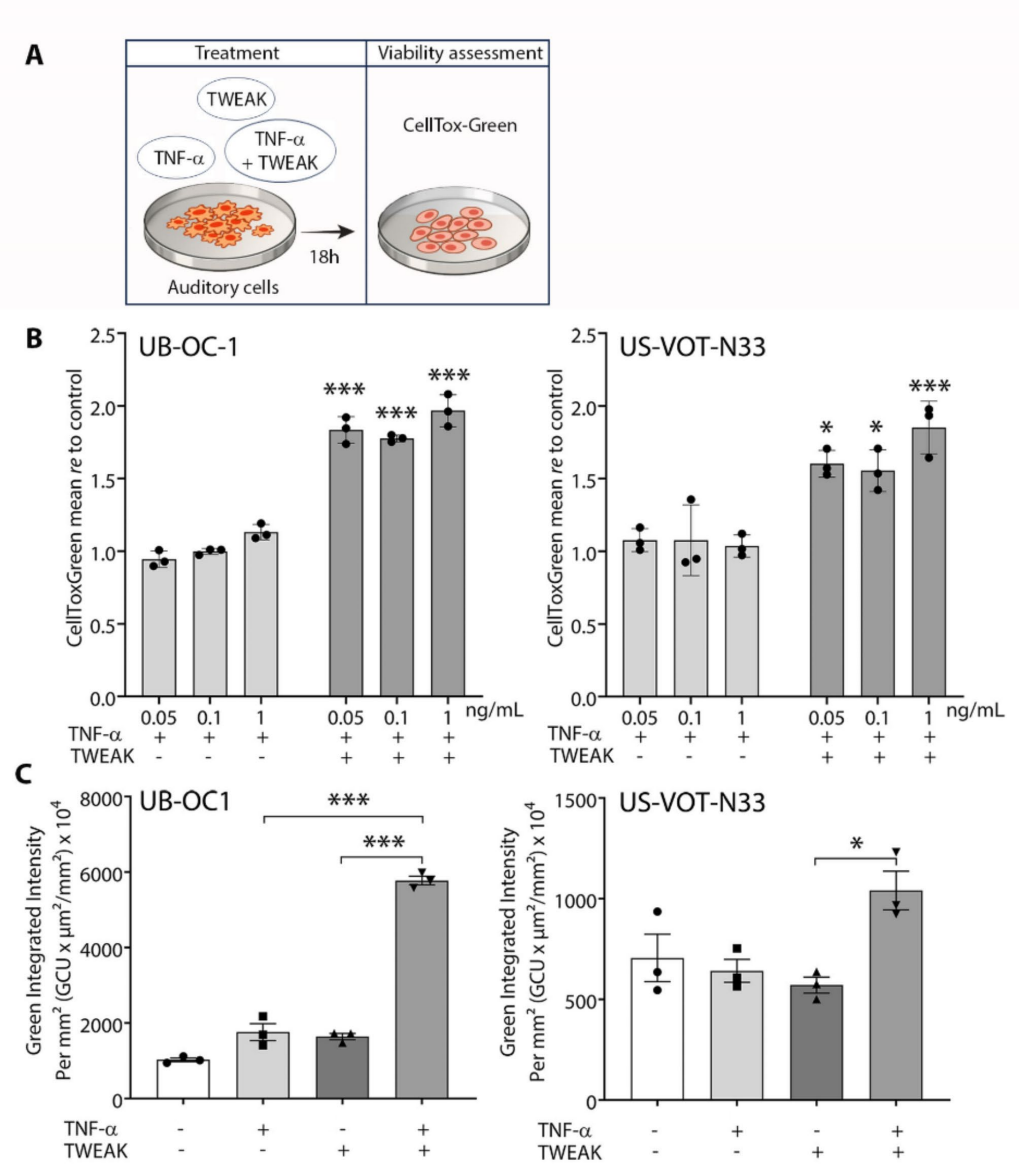
TWEAK and TNF-α synergistically reduce the viability of auditory cells. (**A**) Experimental paradigm to assess the synergistic cytotoxic effects of TWEAK and TNF-α on auditory cells. (**B**) TWEAK and TNF-α co-treatment within the concentration range corresponding to their VS-secreted levels (TWEAK: 50 ng/mL; TNF-α: 0.05, 0.1, and 1 ng/mL) induced cell death in UB-OC1 and US-VOT-N33 cell lines, representing pro-sensory precursors of hair cells and neuroblasts, respectively. Cell death was using CellTox Green Dye staining with live cell imaging and expressed as a mean of fluorescence intensity relative to control-untreated cells. (**C**) TWEAK and TNF-α had a synergistic cytotoxic effect on auditory cells when exposed to equal concentrations of cytokines (50 ng/mL). Cell death is expressed as green integrated intensity per mm². *P < 0.05, **P < 0.01, ***P < 0.001, two-way ANOVA followed by Bonferroni’s multiple comparisons test (**B**), one-way ANOVA followed by Bonferroni’s multiple comparisons test (**C**). Each data point represents technical replicates; data are shown as mean ± s.e.m. GCU, green calibrated unit; VS, vestibular schwannoma

Next, we assessed whether auditory cells are sensitive to TWEAK and TNF-α synergistic cytotoxicity. Mouse cell lines representing pro-sensory precursors of hair cells (UB-OC1) and auditory neuroblasts (US-VOT-N33), were exposed to TWEAK and TNF-α at constant and variable concentration ratios. Similarly to what was observed for the L929 cells, quantification of cell death using CellTox Green staining showed that TWEAK significantly amplified TNF-α cytotoxicity at VS-secreted levels in both UB-OC1 and US-VOT-N33 cell lines (Fig. 9B). A significant synergistic cytotoxic effect was again detected when the cells were co-treated with TWEAK and TNF-α at a constant concentration ratio (50 ng/mL of both cytokines) (Fig. 9C).

## Discussion

This study identified and validated TNF-α and TWEAK as effector molecules that synergistically contribute to VS-induced SNHL. This finding emerged from the systematic profiling of immune-related factors secreted by VS tumors, complementing and corroborating our prior study identifying VS-secreted factors detectable in patients’ blood [27] (Additional file 1: Fig. S10), as well as mechanistic studies using auditory cell lines in vitro and a mouse model of VS in vivo.

Specifically, in this study, we identified 47 cytokines, chemokines, and growth factors secreted by VS tumor tissue, 17 of which were significantly elevated compared to the levels detected in healthy nerve tissue. In Vasilijic et al. (2023), which included over 120 VS patients, we identified 13 factors that were significantly elevated in patients’ plasma as compared to healthy individuals without VS [27]. Eight of these factors—TWEAK, TNF-R2, APRIL, IL-16, IL-2R, MCP-3, eotaxin, and MCP-2—are significantly elevated in both VS secretions and plasma, providing strong suggestive evidence of their role in VS pathogenesis. Importantly, TNF-R2 is a high-affinity receptor for TNF-α, and blood levels of TNF receptors are often used as a proxy for TNF-α levels, particularly in conditions where TNF-α levels are difficult to quantify due to rapid clearance from the bloodstream [42].

Furthermore, we observed a significant positive correlation between tumor-secreted levels and plasma levels for six molecules, suggesting that their elevated plasma levels reflect increased tumor-secretory activity. Notably, TNF-R2, eotaxin, and MCP-3, which have previously been reported to have diagnostic utility in discriminating between VS patients and controls [27], showed a significant correlation between their tumor-secreted and plasma levels, underscoring their potential as biomarkers of VS.

Our finding of elevated levels of TNF-α, IL-2R, CD163, eotaxin, and HGF in VS-CM being significantly associated with hearing impairment in patients with VS suggests that inflammatory pathways and immune cell activation are key contributors to VS-induced hearing loss. This aligns with our previous findings, albeit from a smaller sample size, which identified TNF-α as a potential ototoxic molecule secreted by VS [23]. The observed correlation between increased levels of IL-2R and CD163, representing -biomarkers of T-cell and macrophage activity, respectively [43, 44], and worsened hearing emphasizes the role of these immune cells as potential sources of VS-secreted factors that contribute to hearing loss. This finding is further supported by our recent study demonstrating that VS-associated macrophages secrete a similar panel of cytokines, chemokines, and growth factors as VS cells, implicating them as an important source of the VS secretome [29]. Additionally, the association of elevated HGF levels with lower WR scores indicates that HGF not only plays a role in the pathobiology of VS, as we previously demonstrated [45], but also in VS-associated hearing impairment. Indeed, HGF levels must be carefully controlled because too much or too little HGF is associated with hearing loss in mice and humans [46, 47]. Given our findings that targeting the HGF/cMET signaling pathway could offer an adjuvant therapy to modulate VS growth [45], partially silencing this pathway may also provide a therapeutic benefit for hearing protection in VS patients. Furthermore, the identification of eotaxin as a factor associated with hearing deterioration in VS patients suggests that this chemokine, known for its role in eosinophilic otitis media [48], may also contribute to hearing loss via mechanisms related to sterile inflammation. Eotaxin has been implicated in the pathogenesis of various neurological disorders, potentially through mechanisms involving oxidative stress and microglial activation, leading to glutamate-mediated neurotoxicity [49].

Our analysis of the relationship between tumor size and tumor secretions revealed that larger tumors were associated with higher levels of TNF-α, TNF-R2, IL-1α, IFN-α, MIP-1β, and IL-21. The association between TNF-α and tumor growth has been reported across various tumor types, due to the role of TNF-α in promoting tumor cell survival, proliferation, and the creation of an immunosuppressive microenvironment that fosters tumor progression [50]. TNF-dependent immune suppression primarily involves the activity of regulatory T (Treg) cells and myeloid-derived suppressor cells during chronic inflammation [51, 52]. Additionally, TNF-R2—shown in our study to correlate with VS tumor size—plays an important role in TNF-dependent immunosuppression. Membrane-bound TNF-α binds to TNF-R2, which is expressed on the most potent immunosuppressive subset of Treg cells, driving their expansion and activation [53]. Given that shedding of TNF-R2 is considered to reflect TNF-R2 activity [54], we hypothesize that the levels of soluble TNF-R2 detected in our study may be indicative of the extent of Treg cell infiltration to the VS tumor. This hypothesis is supported by a recent weighted gene co-expression network analysis, which identified Treg and natural killer cells as the immune cell types most strongly associated with VS compared to normal nerves [55]. Future studies are recommended to examine the potential relationship between Treg infiltration and VS tumor progression.

Furthermore, a direct role for TNF-R2 stimulation in VS tumor growth is also plausible, as TNF-R2 has been shown to promote tumor cell proliferation through the phosphoinositide 3-kinase/AKT signaling pathway [56], a pathway known to be inhibited by the merlin protein in healthy Schwann cells [57]. TNF-R2 may also collaborate with TNF-α and other proinflammatory cytokines, such as IL-6, to enhance VS tumor growth, as its expression can be jointly driven by TNF-α and IL-6 [58].

We also observed heterogeneity in TNF-α secretion capacity among VS tumors, categorizing them into TNF-α High and TNF-α Low groups. Importantly, tumors with high TNF-α secretion were found to release cytokines and chemokines that correlated with either poor (TWEAK and eotaxin) or better hearing outcomes (LIF, GRO-α, MIP-1 α, MIP-3 α, and IL-1- α). Among these, TWEAK (Tumor necrosis factor-like weak inducer of apoptosis) emerged as a promising synergistic effector molecule. The secreted levels of TWEAK strongly correlated with poor hearing in patients with TNF-α high-secreting tumors and were elevated in patients with non-serviceable hearing.

Together with TNF-α, TWEAK is a member of the TNF ligand family with a well-established physiological role in tissue repair and a pathological role in diseases related to autoimmune and inflammatory conditions [59]. Although many studies have described the pathophysiological role of TWEAK in renal, cardiac, and respiratory diseases as well as different types of cancer [60, 61], TWEAK’s role in the pathophysiology of hearing loss or VS is unknown.

TWEAK is synthesized as a transmembrane type II molecule that can be proteolytically cleaved into its soluble form [59]. TWEAK performs its function primarily by binding to its cognate receptor– fibroblast growth factor-inducible 14 (Fn14), which is mainly expressed in the cells of inflamed tissues [59]. TWEAK-Fn14 interaction leads to activation of the non-canonical NF-kB signaling pathway, and resultant upregulation of many proinflammatory factors, including TNF-α [62], indicating a potential reciprocal relationship between these two cytokines.

Building on our findings that TNF-α contributes to both VS tumor growth and auditory dysfunction, and recognizing the strong correlation between TWEAK secretion and hearing impairment in patients with VS with high TNF-α secretory capacity, we demonstrated for the first time that tumor secretions, specifically TNF-α and TWEAK, can reach the inner ear in a mouse model of VS-induced SNHL. Notably, we detected tumor-secreted *Gaussia princeps* luciferase in the perilymph from the ear on the tumor-bearing side, but not on the contralateral side. Additionally, we found that tumor secretions were present, though at lower levels, in the blood of tumor-bearing mice. Importantly, we showed that TNF-α and TWEAK levels are elevated in the inner ear with impaired hearing on the tumor-bearing side, providing evidence that tumor-secreted TNF-α and TWEAK can reach the inner ear and potentially induce hearing loss in VS. This finding is further supported by the markedly increased levels of TNF-α and TWEAK observed in human perilymph compared to blood from VS patients. Notably, we showed that patients with tumors having higher TNF-α and TWEAK tumor secretion capacity in vitro also had correspondingly elevated levels of these cytokines in their perilymph, suggesting that our in vitro system could predict in vivo levels of these hearing loss-associated biomarkers. Particularly, a comparison of TNF-α levels in the perilymph, tumor-conditioned media, and blood of three VS patients revealed that only the patient with the highest in vitro tumor secretion capacity for TNF-α exhibited detectable TNF-α levels in the perilymph. This observation indicates a potential relationship between the tumor’s secretory capacity and the diffusion of cytokines into the inner ear environment. These findings are consistent with the high variability in TNF-α secretion levels observed in conditioned media. Additionally, our findings align with prior research highlighting the presence of an inflammatory microenvironment in the human cochlea, as revealed through perilymph analyses using multiplex protein arrays and liquid chromatography-tandem mass spectrometry in patients with cochlear implants and vestibular schwannoma [30–32, 63].

Our demonstration that TWEAK enhances TNF-α cytotoxicity in both TNF-α-sensitive L929 cells and mouse auditory cell lines at concentrations measured in human VS secretions, provides compelling evidence for the amplified ototoxicity of these two cytokines when combined. We show for the first time that TWEAK sensitizes auditory cells to TNF-α-induced cell death, and that this synergistic interaction is blocked with aurintricarboxylic acid, a selective inhibitor of the TWEAK-Fn14 signaling pathway. This finding aligns with existing literature reporting that while Fn14 does not directly interact with death domain-containing adaptor molecules, it can substantially modulate the response triggered by TNF-α binding to TNF-R1, redirecting TNF-α/TNF-R1 proinflammatory signaling toward cell death [64].

Our findings supporting the central role of TNF-α in VS-induced SNHL align with previous transcriptomic analyses of VS that highlighted TNF-α in a top-ranked pathway [65, 66]. Additionally, the link between TNF-α and hearing loss is reinforced by numerous studies [67], including those reporting that TNF-α is one of the most upregulated cytokines in the perilymph 6 h after noise exposure [68], acute intracochlear perfusion of TNF-α induces SNHL and synaptic degeneration [69], and polymorphisms in human TNF-α are associated with the risk of noise-related tinnitus [70] and age-related hearing loss [71].

In contrast, the role of the TWEAK/Fn14 axis in auditory dysfunction remains unexplored. Our current findings suggest that studying this pathway could represent a new research direction, especially given recent studies identifying the TWEAK/Fn14 pathway as a top candidate pathway involved in the modulation of inflammation in Meniere’s disease based on immune genotyping [72], and TWEAK’s ability to impair synaptic transmission and plasticity [73]. Future research should aim to elucidate the mechanistic role of TWEAK in hearing loss using in vivo models, with particular emphasis on investigating the potential of TNF-α and TWEAK/Fn14 inhibitors as therapeutic agents.

## Conclusions

Our study highlights the substantial role of TNF-α and identifies TWEAK as a previously unrecognized effector molecule in VS-induced hearing loss, which amplifies TNF-α ototoxicity. These findings provide a foundation for future research aimed at developing targeted therapies to mitigate hearing loss in affected patients with VS.

### Limitations of this study

While this study provides valuable insights into the molecular mechanisms underlying VS-induced hearing loss, several limitations should be acknowledged. First, the high variability in TNF-α secretion levels in the overall VS cohort highlights the need for larger sample sizes in future research to ensure more robust statistical analyses. Although TNF-α secretion was numerically elevated in conditioned media from VS compared to that from GAN, the difference was not significant. This could be related to the limited number of samples for analysis and/or high variability in TNF-α secretion levels. A significant difference in TNF-α levels was observed between a subgroup of samples with high TNF-α secretion capacity and GAN (*Padj* < 0.001; data not shown); however, the smaller sample size of the TNF-α High group, due to the rarity of VS patients with high TNF-α secretion capacity, remains a limitation. Second, this study used conditioned media, which reflects the secretory capacity of total tumor tissue. Variability in the immune cell composition of the tumor microenvironment between patients may have contributed to the observed differences in TNF-α secretion levels. The specific cellular sources of TNF-α and TWEAK, as well as the mechanisms driving their secretion, remain unclear. Recent findings from our group suggest that tumor tissue exhibits a greater capacity for TWEAK secretion compared to isolated schwannoma cells and tumor-associated macrophages, pointing to a possible role for other tumor-resident cells [29]. Furthermore, interactions between schwannoma cells and tumor-associated macrophages may be critical for TWEAK secretion and require further investigation. Third, while the primary analyses were adjusted for multiple comparisons, associations in exploratory analyses were assessed with Spearman’s correlation coefficients and should, therefore, be interpreted with caution. Finally, the rarity of VS underscores the importance of multi-institutional collaborations for patient recruitment in future studies. Expanding sample sizes through such efforts would enhance the statistical power and generalizability of findings, ultimately providing a deeper understanding of the mechanisms driving hearing loss in VS.

## Materials and methods

### Study population and specimen collection

From 07/2015 to 04/2021, VS tissue and blood were prospectively collected from patients undergoing resection, at Massachusetts Eye and Ear (MEE) in Boston, MA, for unilateral, sporadic VS that had not been previously resected or irradiated. Control GAN tissue was obtained from patients undergoing neck dissections or parotidectomies, during which this nerve is routinely sacrificed. Of 109 enrolled VS patients, 98 met all inclusion criteria and were included in the analyses (CONSORT diagram in Additional file 1: Fig. S1). For comparisons, 35 normal GAN tissue samples were used as controls in some experiments. Human specimens and data collection were approved by the Institutional Review Board of the Massachusetts General Hospital (MGH) and MEE, and all procedures were in accordance with the Helsinki Declaration of 1975.

### Clinical data

Clinical and demographic data were extracted from patient charts, operative reports, pathology reports, and pre-operative radiographic imaging as previously described [25, 27]. The variables assessed included patient age at the time of tissue collection, pre-surgical tumor volume determined via high-resolution contrast-enhanced T1-weighted brain magnetic resonance imaging (MRI) using the internal auditory canal protocol [74]; and pre-surgical pure-tone audiometric threshold and WR scores. The MRI and hearing tests were the ones closest to the resection date, typically within three months. WR score was defined as the percentage of correctly identified spoken monosyllabic words from a list read at 70 dB or at the level where a patient’s speech intelligibility curve plateaus. Pure-tone average (PTA) was calculated from pure-tone audiometric thresholds at 0.5, 1, 2, and 3 kHz. Hearing groups were categorized according to the American Academy of Otolaryngology-Head and Neck Surgery (AAO-HNS) Hearing Classification Guidelines [75]. Serviceable hearing (SH) was defined as WR > 50% and PTA < 50 dB (AAO-HNS class A and B). Patients were classified as having non-serviceable hearing (NSH) if their hearing fell into AAO-HNS class C and D. Tumor resection was considered gross total resection (GTR) if the tumor was completely removed or if a small remnant no greater than 5 × 5 × 2 mm was left to preserve nerve integrity [76]. Otherwise, it was defined as subtotal tumor resection (STR).

### Preparation of tissue-conditioned media

Surgically resected VS and GAN tissues were cleansed of necrotic sections and rinsed three times with phosphate-buffered saline (PBS) (Millipore Sigma). Small tissue fragments were placed into sterile Eppendorf tubes, weighed, and immersed in DMEM media (Thermo Fisher Scientific) without serum and antibiotics, maintaining a ratio of 10 mg of tissue per 100 µL of media [77]. The samples were incubated in culture media at 37 °C in a humidified atmosphere with 5% CO2 for 72 h. The conditioned media was then collected and centrifuged at 300 g for 10 min to eliminate cellular debris and stored at -80 °C until further analysis.

### Isolation and preparation of human plasma and perilymph samples

The collected blood was stored in EDTA vacutainer tubes (Becton Dickinson, NY, US) and kept at 4˚C without freezing until the processing. On the day of collection, the whole blood samples were centrifuged at 2000 g for 10 min at 4 °C. After separation, the plasma was spun at 2000 g for 5 min at 4 °C and filtered through 0.2 μm filter units (MF-Millipore MCE membrane, SLAA033SB; Millipore, Burlington, MA, US). The plasma samples were stored at -80 °C until further use.

Perilymph samples from VS patients were collected during clinically indicated translabyrinthine craniotomies for resection of VS as previously described [78, 79]. The procedure involved thinning the bone of the lateral semicircular canal to expose to membranous labyrinth and collecting approximately 1 µl of perilymph with a 28-gauge needle. The fluid was flushed from the needle using 200 µl of sterile PBS and immediately stored at − 80 °C.

### Measurement of secreted factors in human samples

A comprehensive analysis of 67 immune-related factors was conducted in 55 patients and 20 controls. Due to the wide range of detected values, TNF-α was specifically analyzed in an expanded cohort of 91 patients and 35 controls. Additionally, CD163 and S100B levels were assessed in 67 and 54 patients, respectively.

Luminex, and ELISA assays were conducted as described in detail below. Soluble factors detected in the tumor-conditioned media of > 60% of samples from VS patients were selected for the analysis. The list of analyzed secreted factors is in Additional file 1: Table S1.

#### Luminex assays

Simultaneous multiplex profiling of 65 immune-related factors composed of cytokines, chemokines, and growth factors was performed using a customized multiplex bead-based immunoassay - Immune Monitoring 65-Plex Human ProcartaPlex™ Panel (#EPX650-10065-901; ThermoFisher Scientific, Waltham, MA, US). The levels of TNF-α were assessed by Human TNF alpha High Sensitivity Simplex ProcartaPlex Kit (#EPXS010-10223-901; ThermoFisher Scientific). S100B was detected using a S100B Human ProcartaPlex™ Simplex Kit (#EPX010-12339-901; ThermoFisher Scientific). The assays were performed according to the manufacturer’s instructions. The fluorescence-based signal was acquired on the Magpix instrument (Luminex, Austin, TX, US), and the values of analytes were calculated using ProcartaPlex Analyst 1.0 Software (ThermoFisher Scientific). The analytes with values above the lower limit of quantification in more than 60% of all samples were included in the study (Additional file 1: Table S2). For the accepted analytes, the values exceeding the upper limit of quantification were approximated with the highest concentration representing these limits.

#### ELISA assays

The CD163 ELISA Kit (#EHCD163, Life Technologies, Carlsbad, CA, US) was used to measure soluble CD163 protein levels in the conditioned media. ELISA assays were performed by adhering to the manufacturer’s protocol. Absorbance was measured using the Spectra MAX 190 plate Reader (Molecular Devices, Sunnyvale, CA, US).

### Cell lines and reagents

Mouse *Nf2*^*−/−*^ Schwann cells (gift from Dr. Xandra Breakefield, Massachusetts General Hospital) were maintained in a Schwann cell medium containing 5% fetal bovine serum (FBS), Schwann cell growth supplement, and 1% penicillin and streptomycin (all, ScienCell, Carlsbad, CA, USA) [80].

L929 cells **(**gift from Dr. Alexei Dekterev, Tufts University School of Medicine, Boston, MA, US) were maintained in Dulbecco’s Modified Eagle Medium (DMEM)/F12 medium, 10% FBS, 1% penicillin/ streptomycin (all, Gibco, Thermo Fisher Scientific, Waltham, MA, USA), and 50 µM of 2- mercaptoethanol (Sigma-Aldrich, St. Louis, MO, USA) at 37 °C and 5% CO₂.

UB-OC1 cells (Ximbio, London, UK) were maintained in Minimum Essential Medium (MEM) GlutaMAX™ Supplement (Thermo Fisher Scientific, Waltham, MA, USA), 10% FBS, 50 U/mL of recombinant IFN-γ, (R&D Systems, Minneapolis, MN, USA) and 50 µg/ mL of Normocin (InvivoGen, San Diego, CA, USA) at 33 °C and 5% CO₂. Differentiation of cells was performed at 39 °C in the same culture media but without IFN-γ.

US-VOT-N33 (Ximbio, London, UK) cells were maintained in Neurobasal medium (Gibco, Thermo Fisher Scientific, Waltham, MA, USA) supplemented with 0.5 mM L-glutamine (all, Gibco, Thermo Fisher Scientific, Waltham, MA, USA), 2.5% FBS, 50 U/mL of recombinant IFN-γ, and 50 µg/ mL of Normocin at 33 °C and 5% CO₂.

### Animals

Wild-type immune-competent FVB/C57BL/6 mice (Jackson Laboratory, Bar Harbor, ME, US) 8–12 weeks old were used in experiments. The animals were kept in our animal care facility with ad libitum access to food and water. Ambient sound pressure levels in our animal care facility are in general under 40 dB SPL and peak noise levels do not exceed 70 dB SPL [81]. All animal procedures were performed following the guidelines of Public Health Service Policy on Humane Care of Laboratory Animals and approved by the Institutional Animal Care and Use Committee of the Massachusetts General Hospital (MGH) and Massachusetts Eye and Ear (MEE).

### Detection of vestibular schwannoma secretions in vivo

Vestibular schwannoma tumor growth was induced in immune-competent FVB/C57BL/6 mice (8–12 weeks old) by injecting a mouse *Nf2*^*−/−*^ tumor cell line infected with lentivirus encoding secreted Gaussia luciferase reporter gene (Gluc), as previously described [82]. Briefly, 1 µl of a tumor cell suspension, containing 2,500 cells, was injected into the cerebellopontine angle (CPA) region of the right hemisphere of each mouse. Sham-operated animals were injected with the same volume of saline in the CPA and used as controls. Twelve to fourteen days post-implantation, blood and perilymph samples were collected. The perilymph (1.5 µL) was collected per ear by following the method as previously described [68, 83]. Samples contaminated with blood were discarded, and only clear perilymph specimens were processed. These samples were diluted in double-filtered PBS (Millipore Sigma, Burlington, MA, USA) and stored at − 80 °C until further use. Blood collection involved obtaining 13 µl of whole blood from a slight nick on the tail veins, which was immediately mixed with 5 µl of 50 mM EDTA to prevent clotting. The samples were centrifuged at 10,000 g for 5 min, and the resulting diluted plasma was transferred to Eppendorf tubes and stored at − 80 °C. Gluc activity was detected using coelenterazine (CTZ, Nanolight Technology, Pinetop, AZ, USA) and quantified with a GloMax 96 Microplate Luminometer (Promega, Madison, WI, USA), as previously described [82, 84].

### Measurement of TNF-α and TWEAK in mouse perilymph and blood

The perilymph and blood from tumor-bearing animals and sham-operated animals were collected 12–14 days after tumor cells were implanted or saline was injected. Mouse TWEAK was detected by a customized Mouse Magnetic Luminex Assay (#LXSAMSM-03, R&D Systems, Minneapolis, MN, USA) according to the manufacturer’s instructions, using equipment and software as described for Luminex assays above. Mouse TNF-α was quantified using V-PLEX Proinflammatory Panel 1 Mouse Kit (#K15048D, Meso Scale Discovery, Rockville, MD, USA), according to the manufacturer’s instructions. Signal detection was performed on the QuickPlex SQ 120 device (MSD). Preanalytical data processing was done using MSD Discovery Workbench software (v4.0.12).

### Audiometric testing in animals

Twelve to fourteen days after *Nf2-/-* tumor cells implantation, the auditory brainstem responses (ABRs) were measured as described earlier [82]. Briefly, animals were anesthetized via intraperitoneal injection of ketamine (0.1 mg/g) and xylazine (0.02 mg/g). The tympanic membrane and the middle ear were microscopically examined for signs of otitis media. All animals had well-aerated middle ears. ABRs were recorded between subdermal needle electrodes: positive in the inferior aspect of the ipsilateral pinna, negative at the vertex and ground at the proximal tail. The responses were amplified (10,000X), filtered (0.3–3.0 kHz) and averaged (512 repetitions) for each frequency and sound level. Custom LabVIEW software for data-acquisition was run on a PXI chassis (National Instruments Corp). For each frequency, the auditory threshold was defined as the lowest stimulus at which repeatable peaks could be observed on visual inspection. In the absence of an auditory threshold, a value of 85 dB was assigned (5 dB above the maximal tested level).

### In vitro cytotoxicity

Cell lines were expanded in a 96-well plate (USA Scientific, Ocala, FL, USA) under the following appropriate permissive conditions before treatment with recombinant cytokines. After reaching 80% of confluence, the cells were treated with human recombinant TNF-α and TWEAK (both R&D Systems, Minneapolis, MN, USA) for 24 h in DMEM/F12 medium and 10% FBS, at 37 °C and 5% CO₂. The cytotoxic effect was assessed based on the reduction of metabolic activity (MTT assay) and induction of necroptotic cell death by quantification of lactate dehydrogenase activity in cell culture supernatants (LDH assay) and assessment of the cell membrane integrity (CellTox Green Cytotoxicity assay**)** as described below.

#### MTT assay

Following incubation with TNF-α and TWEAK, the cells were gently washed with warm PBS. Subsequently, 100 µL of 0.5 mg/mL MTT [3-(4,5-dimethylthiazol-2-yl)-2,5-diphenyltetrazolium bromide] solution (Molecular Probes, Eugene, OR, USA) prepared in serum-free DMEM/F12 medium was added to each well. The cells were then incubated for 4 h at 37 °C in a 5% CO₂ atmosphere. Formazan crystals formed during this incubation were solubilized by adding 100 µL of SDS-HCl solution (10% SDS in 0.01 M HCl; Sigma-Aldrich, St. Louis, MO, USA) to each well and incubating for an additional 16–18 h. Absorbance was measured at 570 nm using a SpectraMax iD3 Multi-Mode Microplate Reader (Molecular Devices, LLC, San Jose, CA, USA). The metabolic activity of the cytokine-treated cells was expressed relative to that of untreated control cells.

#### LDH assay

Colorimetric Lactate Dehydrogenase (LDH) Assay Kit (Abcam, Cambridge, MA, USA) was used according to the manufacturer’s instructions. Briefly, culture supernatant (50 µL) from TNF-α and TWEAK-treated cells was mixed with 50 µL of Reaction Mix. Absorbance was measured at 450 nm using a SpectraMax iD3 Multi-Mode Microplate Reader. The LDH activity was quantified according to the detected levels of NADH and was expressed relative to untreated control cells.

#### CellTox green cytotoxicity assay

Necroptotic cell death was evaluated using CellTox Green Dye (Promega, Madison, WI, USA), a non-fluorescent dye that fluoresces upon binding to DNA in cells with compromised membranes. Briefly, immediately after the addition of TNF-α and TWEAK, 0.1% (v/v) CellTox Green Dye was added to the wells of a 96-well plate. The cells were then incubated at 37 °C with 5% CO2 and imaged using the Incucyte^®^ S3 Live-Cell Analysis System (Essen BioScience, Ann Arbor, MI, USA). Images were captured hourly for 24 h to monitor green fluorescence, indicating dead cells, at 10x magnification. The images were analyzed using IncuCyte Basic Analysis Software (Sartorius, Ann Arbor, MI, USA), and the results are presented as the mean green fluorescence intensity, reflecting cells with damaged membranes.

### Synergy testing

L929 cells (1–2 × 104 cells per 100 µL) were maintained in 96 well plates as described above for 24 to 48 h, until reaching 60–80% of confluency. The cells were treated with TWEAK and TNF-α for 18 h using a 7 × 7 dose-response matrix in duplicates, combining concentrations ranging from 0.01 ng/mL to 100 ng/ mL. Cell viability was assessed using the MTT assay described above. Single drug dose-response curves, combination indexes, and synergy scores were calculated using CompuSyn software2 and SynergyFinder (version 3.0) web-based application for analyzing drug combination dose-response matrix data (https://synergyfinder.fimm.fi) [41].

### Statistics

Generalized least squares (GLS) model was used for the following patient group comparisons: (1) VS patients versus controls, (2) VS-SH patients versus VS-NSH, (3) secretion capacity of tumor tissue from VS patients who underwent GTR versus STR, and (4) TNF-α High vs. TNF-α Low tumor secretion capacity. The regression model was used as previously described [27]. Briefly, all secreted factors were natural log transformed prior to modeling. To compare secreted factor levels the following GLS model was used: ln < molecule > = *b0* + *b1*Sex + *b2*Age + *b3S*H + *b4*NSH. The molecule in the formula refers to tested secreted factors, while b0, b1, b2, b3, and b4 are regression coefficients representing the change in natural log-transformed concentrations of prospective secreted factors to a one-unit change in the respective independent variable (sex, age, SH, and NSH). For mutual comparison of SH and NSH groups, the GLS model was extended to control the subject’s tumor volume. For all comparisons, *p* < 0.05 was considered statistically significant. P values were corrected for multiple comparisons (and called *Padj*) using the Benjamini-Hochberg false detection rate (FDR) procedure. The procedure was applied for multiple comparisons in several analyses, including the comparison of cytokine levels between tumor-conditioned media (VS) and media conditioned by normal tissue (control), between tumor tissues from vestibular schwannoma patients with serviceable hearing (SH) and non-serviceable hearing (NSH), between the TNF-α High and TNF-α Low groups, and between tumor tissues from gross total resections (GTR) and subtotal resections (STR).Differences in age across groups were analyzed using one-way ANOVA with Dunn’s multiple comparisons test. Differences in tumor volume and PTA were analyzed using the Mann-Whitney t-test. Differences in WR were analyzed using *N* − 1 chi-squared test for the comparison of two proportions expressed as a percentage. Ratios of secreted factors released in tumor tissue- and normal tissue-conditioned media, as well as ratios of factors secreted by tumors from patients with SH and NSH were calculated by dividing their respective average concentrations (pg/mL) after outlier removal. Average concentrations of secreted factors were determined from groups of VS patients and controls matched for sex and age.

Spearman’s correlation coefficient was employed to evaluate the relationships between tumor-secreted factors and clinical parameters in VS patients, as well as between the levels of tumor-secreted factors in vitro and their corresponding plasma levels. Given the exploratory nature of this analysis, which aimed primarily at identifying potential associations between tumor-secreted factors, hearing loss, and tumor size, formal corrections for multiple comparisons were not applied. This approach is consistent with established guidelines for early-stage research, where the priority is often on sensitivity to detect possible associations, with the understanding that specificity may be of secondary importance at this stage [85].

Prior to conducting correlation analyses, a thorough data quality assessment was performed. Descriptive statistics, including skewness and kurtosis, were calculated to evaluate the distribution of the data (Additional file 1: Table S3). Secreted factors with moderate skewness (− 1 to 1) were deemed suitable for Spearman correlation analysis without further adjustment. For secreted factors with skewness exceeding 1, the Robust Regression and Outlier Removal (ROUT) method in GraphPad Prism software was employed to identify and exclude outliers. The maximum acceptable false discovery rate (Q parameter) was set to 0.1%, enabling the detection of the most extreme outliers while minimizing the risk of false positives. The impact of outlier removal on skewness and kurtosis of data distribution is shown in Additional file 1: Table S4.

#### Statistical software

Comparisons of demographics, tumor volume, and hearing loss between groups were performed with GraphPad Prism (v9.3.1; GraphPad Software, La Jolla, CA, US) and MedCalc^®^ Statistical Software (v20.109; MedCalc Software Ltd, Ostend, Belgium). Models were estimated, and graphs were generated using R (v3.6.3; R Foundation, Vienna, Austria). The relationship between tumor-secreted factors in vitro and plasma level was assessed by Spearman correlation analysis using GraphPad software.

## Electronic supplementary material

Below is the link to the electronic supplementary material.

Supplementary Material 1

## Data Availability

All data needed to evaluate the conclusions in the paper are present in the paper and/or the supplementary files.

## Abbreviations

ABR: Auditory brain stem response
ATA: Aurintricarboxylic acid
CI: Combination index
CM: Conditioned media
CPA: Cerebellopontine angle
dB: Decibel
DRI: Dose-Reduction Index
Fa: Fractional inhibition
GAN: Great auricular nerve
GAN-CM: Great auricular nerve- conditioned media
GCU: Green calibrated unit
GTR: Gross total resections
Hz: Hertz
LDH: Lactate dehydrogenase
LLOQ: Lower limit of quantification
MRI: Magnetic resonance imaging
MTT: [3-(4,5-dimethylthiazol-2-yl)-2,5-diphenyltetrazolium bromide
NA: Not available
NF2: Neurofibromatosis 2
NSH: Non-serviceable hearing
Padj: Adjusted P value corrected for multiple comparisons
PTA: Pure tone average
RLU: Relative light unit
SD: Standard deviation
SH: Serviceable hearing
SPL: Sound pressure level
STR: Subtotal resections
ULOQ: Upper limit of quantification
VS: Vestibular schwannoma
VS-CM: Vestibular schwannoma-conditioned media
VS-NSH: Vestibular schwannoma patients with non-serviceable hearing
VS-SH: Vestibular schwannoma patients with serviceable hearing
WR: Word recognition
