# Correction: Immune profiling of human vestibular schwannoma secretions identifies TNF-α and TWEAK as cytokines with synergistic potential to impair hearing

**DOI:** 10.1186/s12974-025-03484-6

**Published:** 2025-08-01

**Authors:** Sasa Vasilijic, Richard Seist, Zhenzhen Yin, Lei Xu, Konstantina M. Stankovic

**Affiliations:** 1 Department of Otolaryngology– Head and Neck Surgery, Stanford University School of Medicine, 801 Welch Rd, Palo Alto, 94304 Stanford, CA USA; 2 Edwin L. Steele Laboratories, Department of Radiation Oncology, Massachusetts General Hospital, Harvard Medical School, Boston, MA USA; 3 Department of Neurosurgery, Stanford University School of Medicine, Stanford, CA USA; 4 Wu Tsai Neurosciences Institute, Stanford University, Stanford, CA USA

**Correction to: Journal of Neuroinflammation (2025) 22:35**

10.1186/s12974-025-03364-z.

In this article [1], the panel 3B of Fig. 3 and the panel 5E of Fig. 5 were published incorrectly.

 Specifically, in panel 3B, the last scatterplot intended to show the correlation between plasma and tumor-secreted values for MCP-2 was mistakenly replaced with a duplicated scatterplot for eotaxin, due to an inadvertent layer overlap during figure preparation.

In panel 5E, the numerical values for tumor size and the y-axis title (“tumor size (cm³)”) are missing due to an unintended overlap of layers during the figure preparation process. The corrected versions of Fig. 3 and Fig. 5 are shown below.

Additionally, on page 6, the Spearman correlation coefficient and p-value reported for eotaxin were inadvertently retained from an earlier analysis that included outliers. These values have been corrected based on a revised analysis that excludes outliers. Accordingly, the following sentence: “Eotaxin was again associated with poor hearing, yet with a stronger correlation than in the unseparated cohort (PTA: r = 0.556, P = 0.022, versus r = 0.292, P = 0.042) (Figs. 4B, and 6B).” is revised to: “Eotaxin was again associated with poor hearing, yet with a stronger correlation than in the unseparated cohort (PTA: r = 0.556, P = 0.022, versus r = 0.339, P = 0.015) (Figs. 4B, and 6B).”

Correct Fig. 3.

**Fig. 3:**
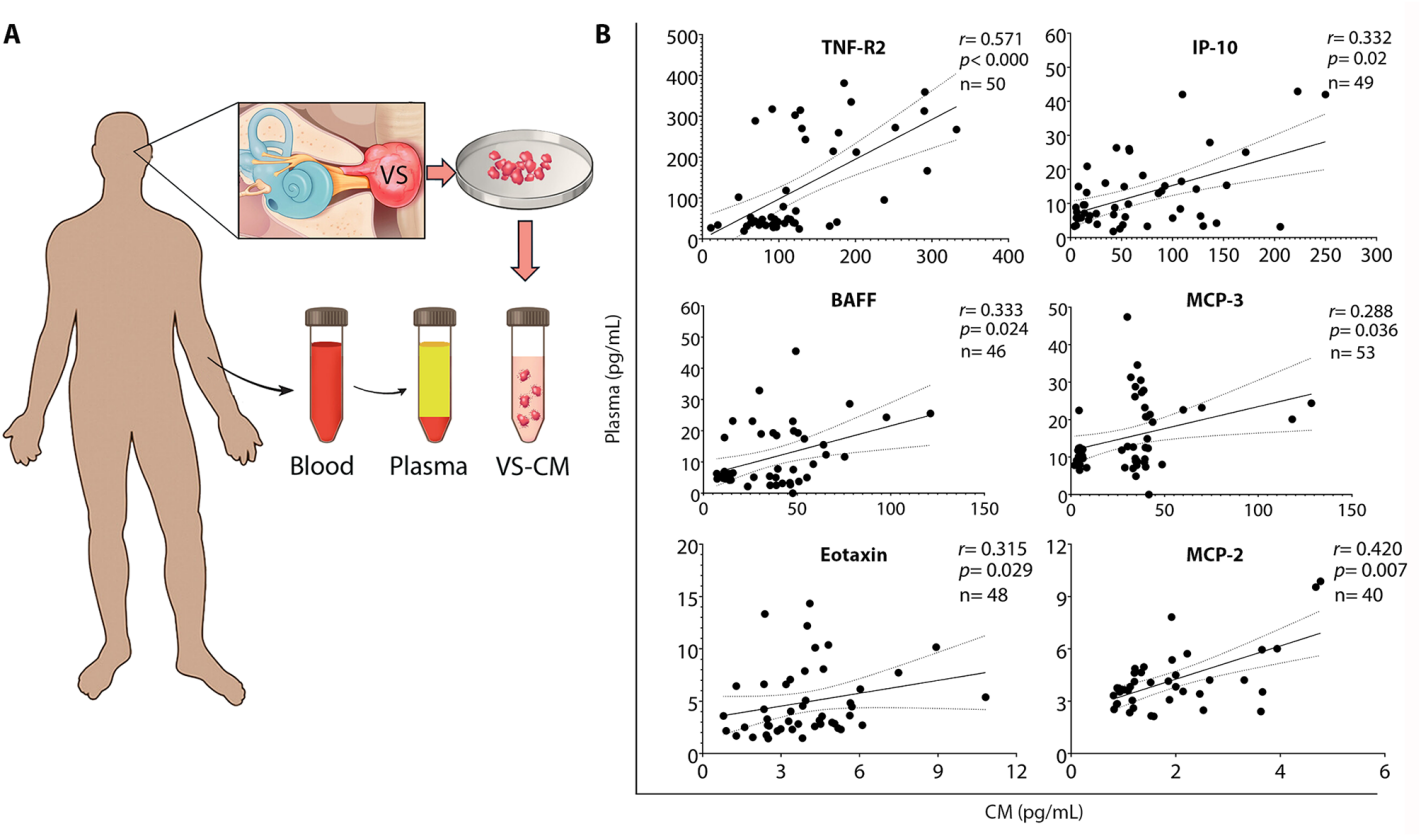
Systemic levels of secreted factors correlate with tumor-secreted capacity in vitro. (**A**) Study design to analyze the correlation between the levels of secreted factors in plasma and tumor-conditioned media. (**B**) Six out of the 17 secreted factors significantly elevated in vitro positively correlate with their corresponding plasma levels, as analyzed using Spearman’s correlation coefficient ‘r’. The solid line depicts the regression curve, while the dotted lines indicate the 95% confidence intervals of the best-fit line. Each black circle represents a unique VS patient. VS, vestibular schwannoma; CM, conditioned media; VS-CM, vestibular schwannoma- conditioned media

Correct Fig. 5.

**Fig. 5:**
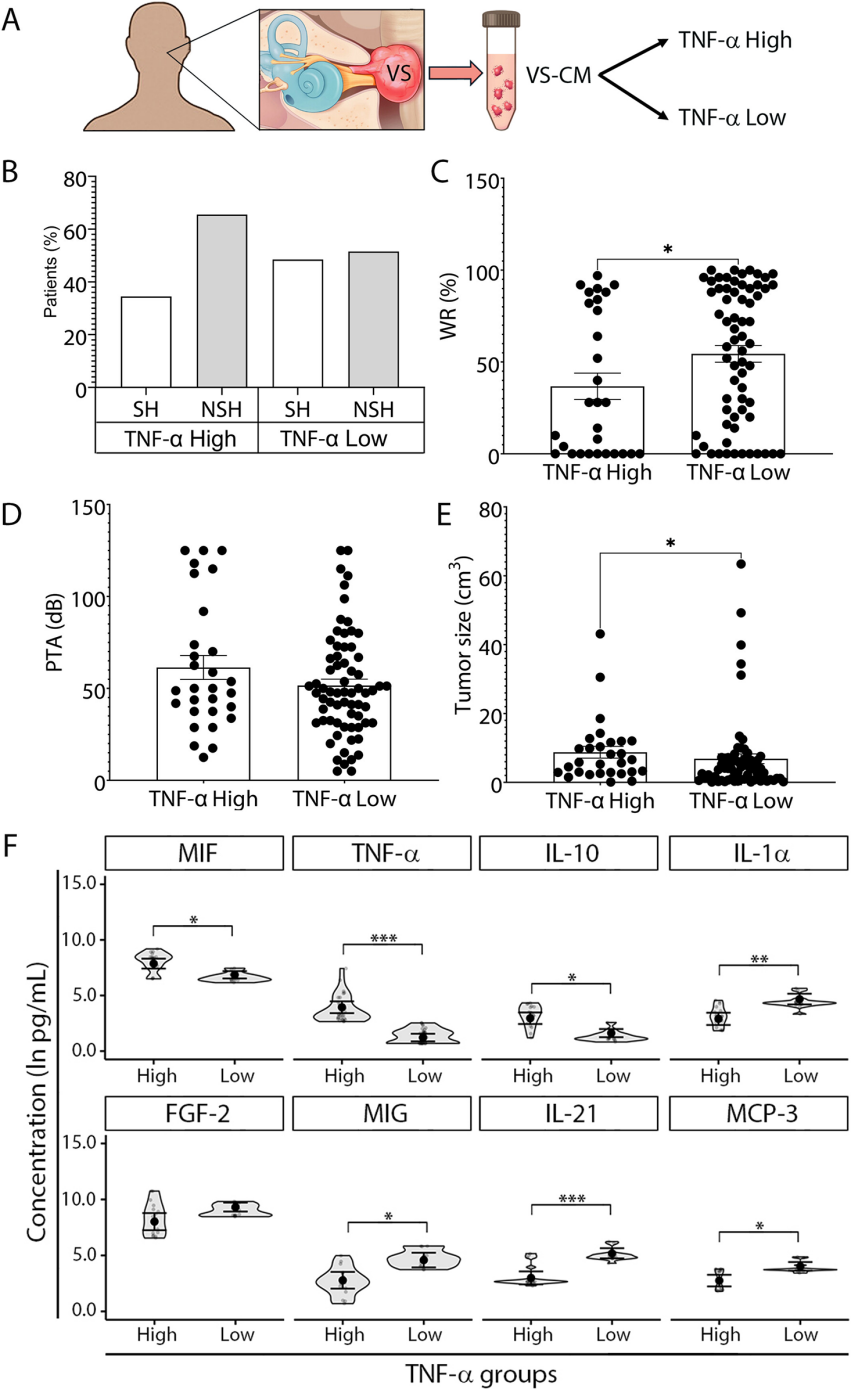
Clinical characteristics and immune factors in VS patients with high versus low TNF-α tumor secretion. (**A**) Study design to compare preoperative clinical characteristics and secreted factors between VS patients with TNF-α high and low tumor-secretion capacity. (**B**) The distribution of patients with serviceable hearing (SH) and non-serviceable hearing (NSH) in TNF-α High (*n* = 29) and TNF- α Low groups (*n* = 69) highlights the higher prevalence of NSH in the TNF-α High group. VS patients were categorized into TNF-α High and TNF-α Low groups based on whether their tumor-secreted TNF-α levels were above or below the average TNF-α levels secreted by normal nerve tissue (Additional file 1: Fig. S7). (**C**, **D**) Comparison of hearing outcomes: patients in the TNF-α High secretion group exhibited worse hearing with lower WR scores than patients in the TNF-α Low secretion group, despite similar PTA values. (**E**) Tumor volume was, on average, larger in the TNF-α High group as compared with the TNF-α Low group. (**F**) Differential expression of immune-related factors: eight out of 47 significantly altered factors between tumor and normal tissue demonstrated significant differences between TNF-α high (*n* = 17–21) and TNF-α low groups (*n* = 37–46). C, D, E, Mann-Whitney two-tailed test. F, Generalized linear mixed effects regression- GLS model. Each data point represents a unique VS patient. The full names of secreted factors are listed in Additional file 1: Table S1. *P < 0.05, **P < 0.01, ***P < 0.001. VS, vestibular schwannoma; VS-CM, vestibular schwannoma-conditioned media; PTA, pure tone average; dB, decibel; WR, word recognition
