# Supplementary material for: Immune profiling of human vestibular schwannoma secretions identifies TNF-α and TWEAK as cytokines with synergistic potential to impair hearing
Source: J Neuroinflammation. 2025 Feb 8;22:35. doi: 10.1186/s12974-025-03364-z (PMC11807327; doi:10.1186/s12974-025-03364-z)
Supplement: Supplementary file 1 — Supplementary Material 1 [file 12974_2025_3364_MOESM1_ESM.docx]

Supplementary Materials for

**Immune Profiling of Human Vestibular Schwannoma Secretions Identifies TNF-α and TWEAK as Cytokines with Synergistic Potential to Impair Hearing**

Sasa Vasilijic *et al.*

*Corresponding author. Konstantina Stankovic, Email: [kstankovic@stanford.edu](mailto:kstankovic@stanford.edu)

**This file includes:**

Figs. S1 to S10

Tables S1 to S4

**
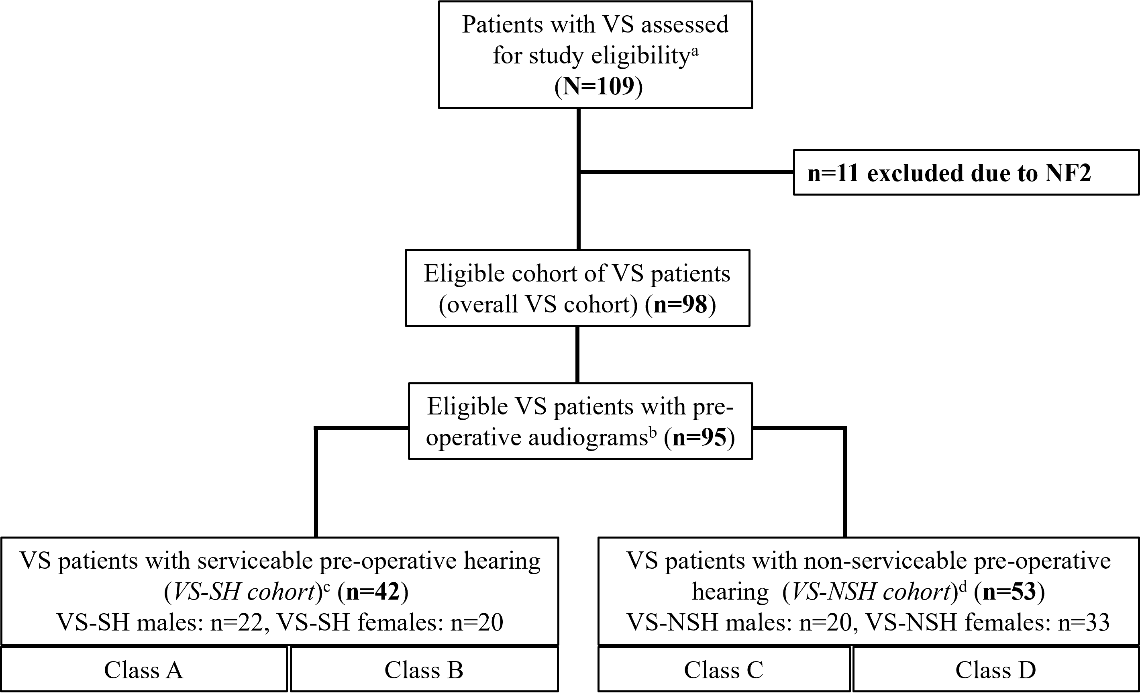
**

Fig. S1. CONSORT flow diagram for the VS cohort

Among the overall cohort of eligible VS patients, subgroups are defined by pre-operative hearing ability based on AAO-HNS guidelines.

Notes: ^a^ Eligible patients have unilateral, sporadic VS that has not been previously resected or irradiated; ^b^ One VS patient has no pre-operative audiogram and two VS patients have missing word recognition scores. ^c^ Serviceable hearing is defined as AAO-HNS Class A and B hearing (PTA ≤ 50 dB and WR score ≥ 50%). ^d^ Non-serviceable hearing is defined as AAO-HNS Class C and D hearing (either PTA > 50 dB or WRS <50%). Abbreviation: VS, vestibular schwannoma; VS-SH, vestibular schwannoma patients with serviceable hearing; NF2, neurofibromatosis 2; VS-NSH, vestibular schwannoma patients with non-serviceable hearing.

**
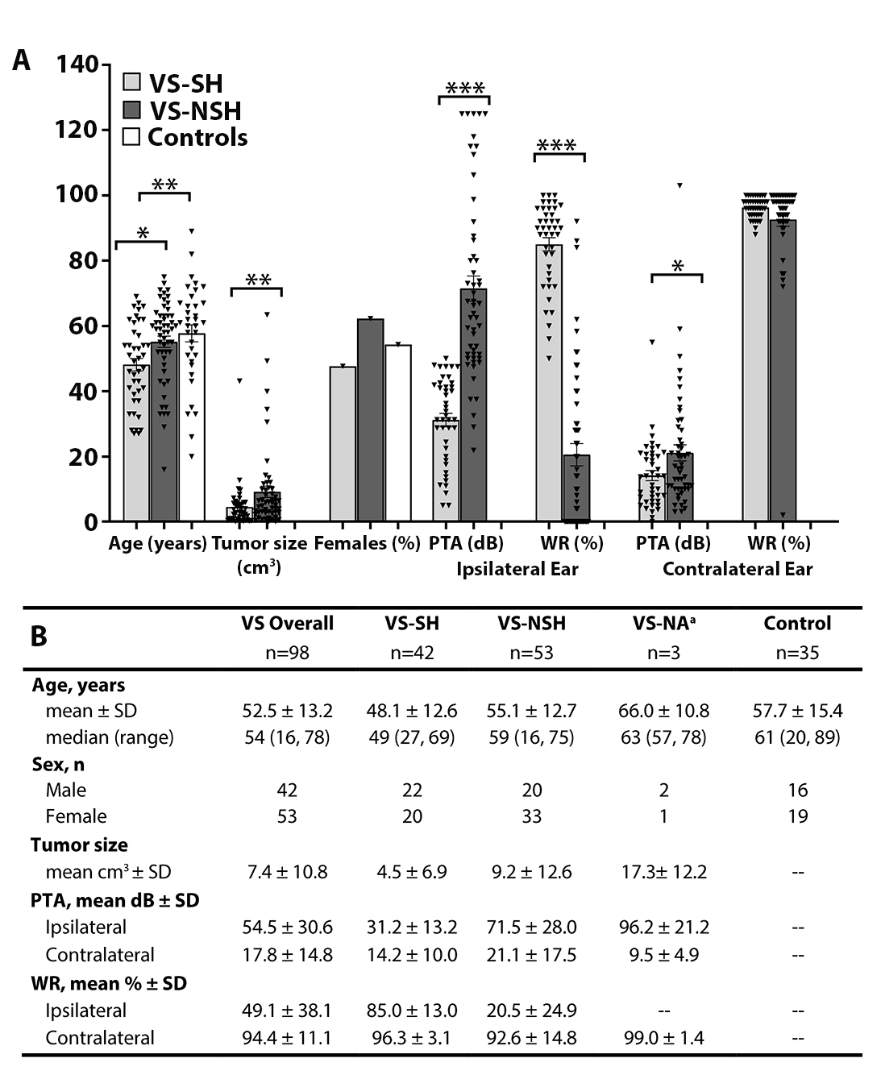
**

**Fig. S2. Demographic and clinical characteristics of the VS patients and controls**

(A) Each data point (triangle) represents a VS patient or donor of healthy nerve tissue; data are shown as means with error bars (s.e.m). (B) Key characteristics of VS patients and controls. Ipsilateral refers to the same side as the tumor. Kruskal-Wallis test followed by Dunn's multiple comparisons test were used for comparing mean age. Mann-Whitney two-tailed test was used for comparisons of tumor size and PTA. N−1 chi-squared test was used for comparison of WR. **P*<0.05, ***P*<0.01, ****P*<0.001. Note: ^a^ VS-NA refers to the small group of VS patients without complete audiometric parameters required for hearing classification (n=3). Abbreviations: dB, decibel; NA, not available; PTA, pure-tone average; SD, standard deviation; VS, vestibular schwannoma; VS-SH, VS patients with serviceable hearing; VS-NSH, VS patients with non-serviceable hearing; WR, word recognition.

**
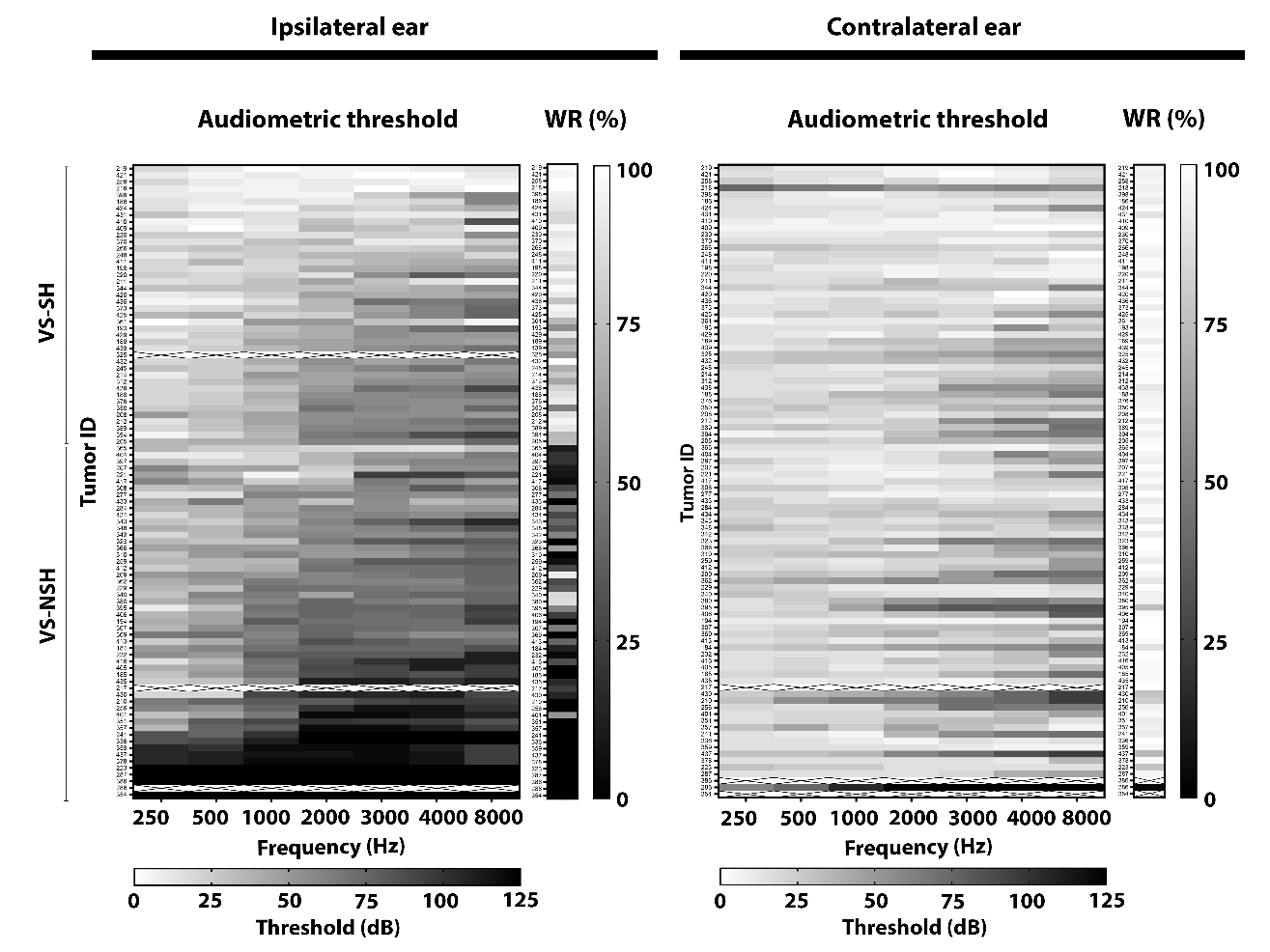
**

**Fig. S3. Hearing characteristics of VS patients**

Abbreviation: dB, decibel; SH, serviceable hearing; Hz, hertz; NSH, non-serviceable hearing; VS, vestibular schwannoma; WR, word recognition.


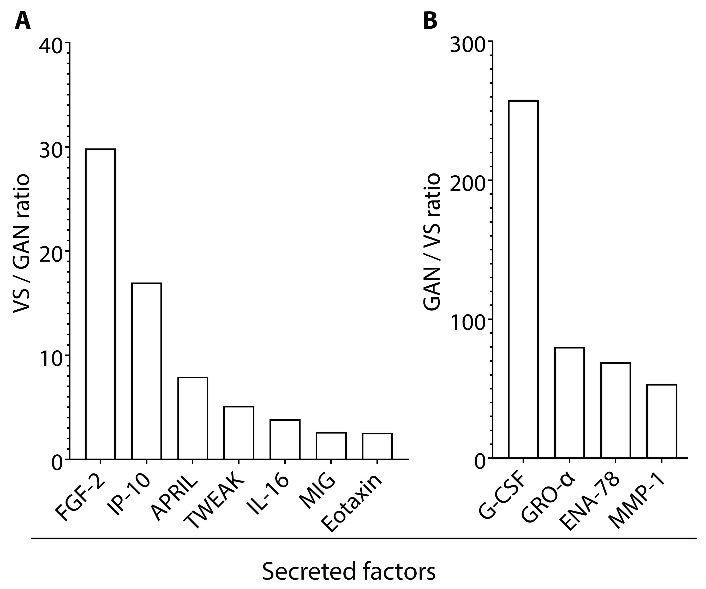


**Fig. S4. Factors most elevated in VS and GAN tissue secretions**

(A) Seven out of 17 significantly elevated tumor-secreted factors are increased by more than 2.5-fold compared to healthy GAN tissue (VS/GAN ratio). (B) Four out of seven significantly elevated factors in healthy GAN tissue are increased by more than 2.5-fold compared to VS tissue (GAN/VS ratio). Ratios are calculated by dividing the average levels of secreted factors from age- and sex-matched groups of VS patients and donors of healthy control GAN tissue. The full names of the secreted factors are provided in Table S1. Abbreviations: VS, vestibular schwannoma; GAN, great auricular nerve.


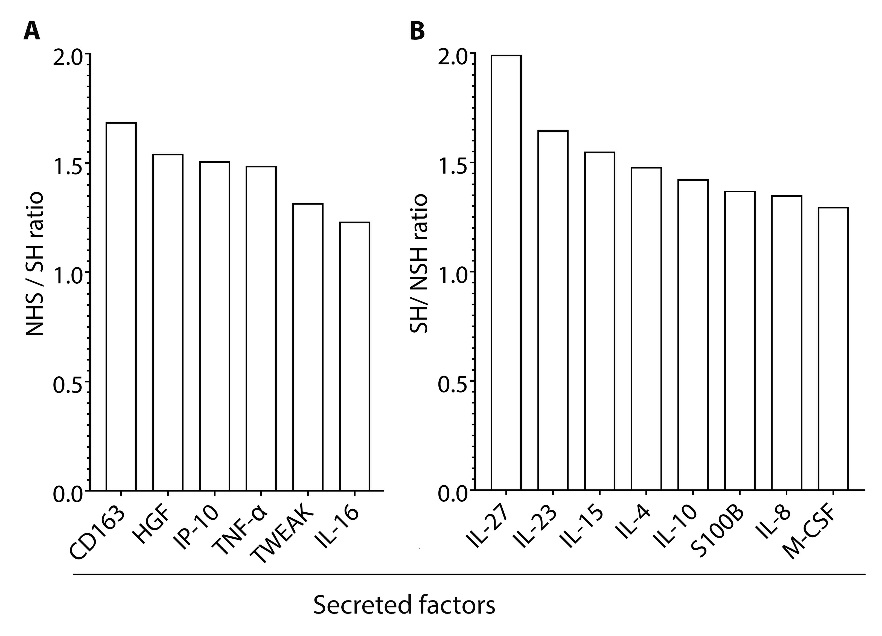


**Fig. S5. Differentially secreted immune factors in VS patients with serviceable and non-serviceable hearing**

(A) Secreted factors significantly elevated in VS-SH compared to VS-NSH tissue (VS-SH/VS-NSH ratio). (B) Secreted factors significantly elevated in VS-NSH compared to VS-SH tissue (VS-SH/VS-NSH ratio). The ratio is calculated by dividing average levels of secreted factors from age and sex-matched groups of VS patients with serviceable and non-serviceable hearing. Secreted factors elevated more than 1.2 times are shown. The serviceable hearing is defined as AAO-HNS Class A and B hearing (PTA ≤ 50 dB and WRS ≥ 50%). Non-serviceable hearing is defined as AAO-HNS Class C and D hearing (PTA > 50 dB or WRS <50%). The full names of secreted factors are listed in Table S1. Abbreviations: SH, serviceable hearing; NSH, non-serviceable hearing.

**

**

**Fig. S6. Secretory capacity of tumor tissue following gross total versus subtotal resection in VS patients**

(A) Tumor tissues from gross total resections (GTR) and subtotal resections (STR) exhibit differential secretion of five cytokines and chemokines, with TNF-α being the only cytokine significantly elevated in tumors that underwent STR. (B) Tumors that underwent STR are significantly larger in size than those removed by GTR. A, **P*<0.05, ***P*<0.01, ****P*<0.001, Generalized linear mixed effects regression- GLS model. Each data point represents a VS tumor tissue. B, **P*<0.05, Mann-Whitney two-tailed test. Each data point represents a VS patient.


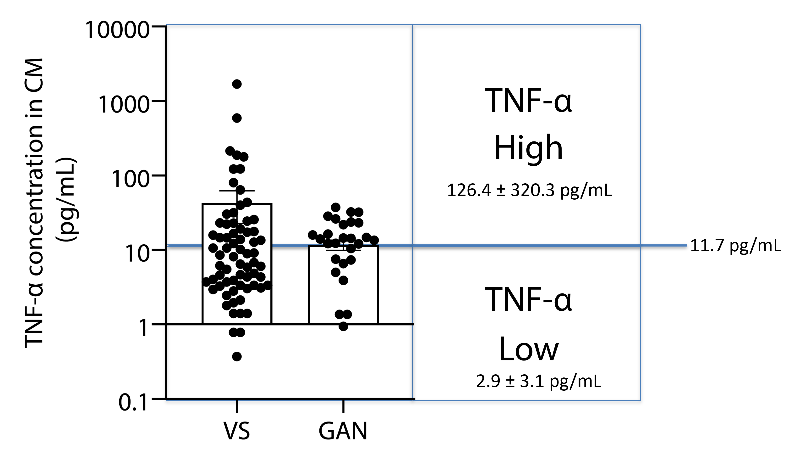


**Fig. S7. VS patients categorized into TNF-α High and Low groups based on tumor tissue secretion capacity**

Ninety-eight VS patients are classified into TNF-α High (n=29) and TNF-α Low (n=69) groups based on whether their tumor-secreted TNF-α levels in VS-CM are above or below the average TNF-α levels secreted by healthy nerve tissue (11.66 pg/mL). Abbreviations: VS, vestibular schwannoma; GAN, great auricular nerve; CM, conditioned media.


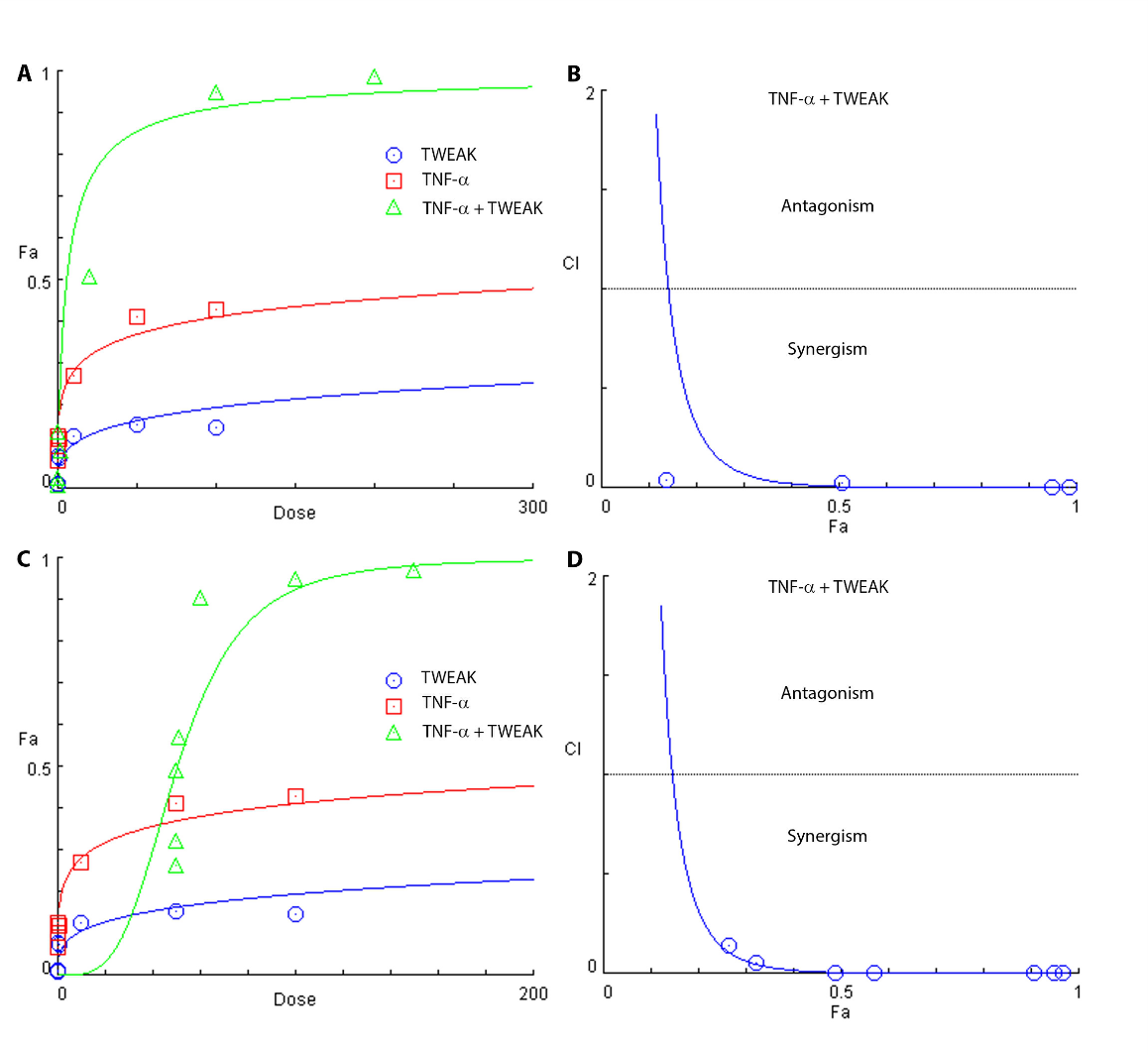


**Fig. S8.** **Synergistic interaction of TWEAK and TNF-α**

(A) Dose-response for L929 cells, calculated using CompuSyn software (Chou-Talalay method), shows that the fractional inhibition of metabolic activity, Fa, is substantially higher for TWEAK and TNF-α combination at iso-concentrations of 10, 50 and 100 ng/mL than for either cytokine alone. (B) Combination index plots for TWEAK and TNF-α combinations demonstrate very strong synergism (CI < 0.1) for iso-concentrations of 10, 50, and 100 ng/mL. However, at iso-concentrations of 1, 0.1, and 0.05 ng/mL, a very strong antagonism (CI>10) is achieved (data not shown above CI =2). The horizontal line at CI = 1 indicates an additive effect. (C) Dose-response curves for L929 cells treated with TWEAK (50 ng /mL) and seven different concentrations of TNF-α (0.01–100 ng/mL) for 18 h. Fa is substantially higher for TWEAK and TNF-α combinations than for either cytokine alone. (D) The combination index plot for TWEAK and TNF-α combinations at different concentration ratios applied to L929 cells demonstrates clear synergism across all tested cytokine concentrations. CI, combination index; Fa, fractional inhibition.


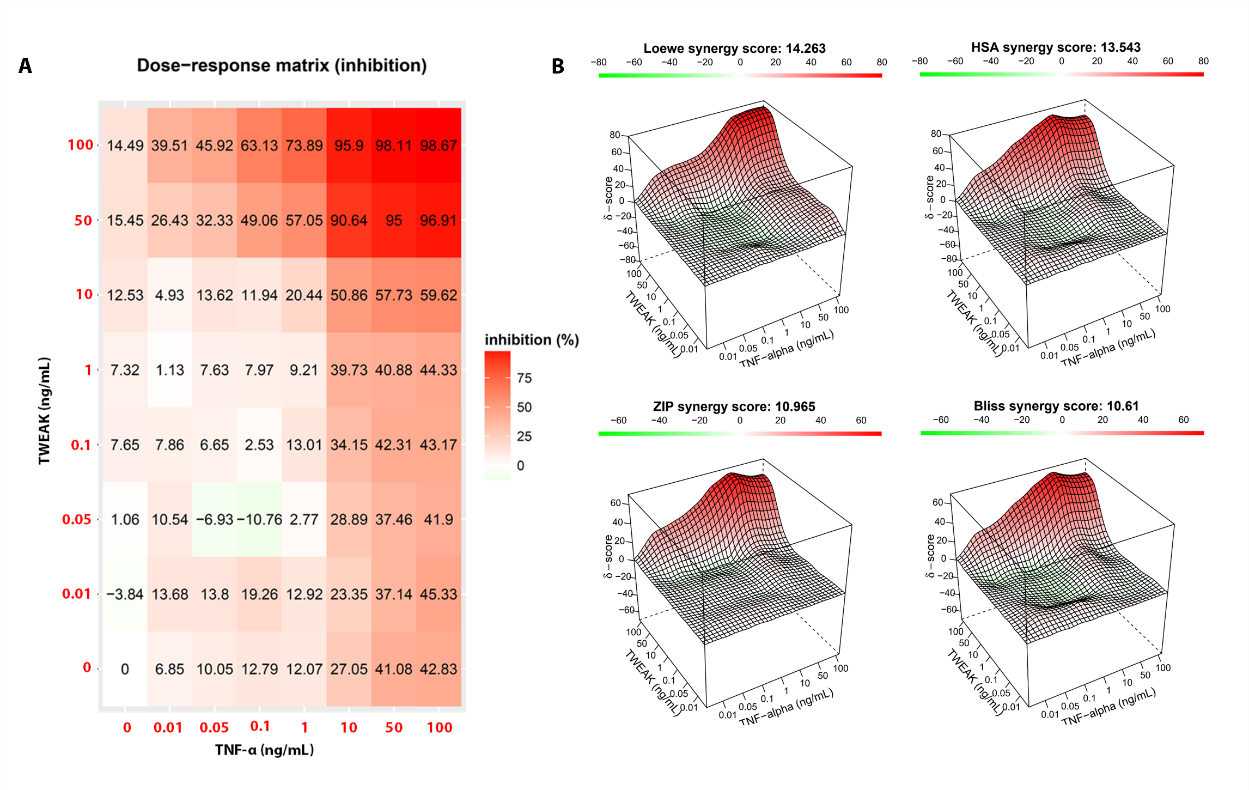


**Fig. S9.** **Quantification of TWEAK and TNF-α synergistic interaction**

L929 cells are treated with TWEAK and TNF-α for 18h using a 7x7 dose-response matrix, including concentrations ranging from 0.01 ng/mL to 100 ng/ mL, spanning the levels released by VS tissue. The synergistic response is determined by quantifying metabolic activity using an MTT assay. The inhibition of metabolic (%) activity is calculated relative to untreated cells. (A) Dose-response matrix heatmap of inhibition of metabolic activity as a function of different cytokine concentrations. (B) Synergy plots of TWEAK and TNF-α combination matrices. 3D synergy maps illustrate synergistic dose regions (highlighted in red). Synergy score (δ-score) higher than 10 is considered indicative of true synergistic interaction. Plots are created using the SynergyFinder web application for analyzing cytokine combination dose–response matrix data [*31*].





**Fig. S10. Summary of VS-secreted molecules and their overlap with blood biomarkers.**

VS-secreted molecules identified in this study and their overlap with the potential blood biomarkers of VS identified in the Vasilijic et al. 2023 study [27].

Table S1. List and full names of assayed immune-related factors

| **Common name** | **Full name** | **Common name** | **Full name** | **Common name** | **Full name** |
| --- | --- | --- | --- | --- | --- |
| APRIL | A proliferation-inducing ligand | IL-21 | Interleukin-21 | MIP-1β | Macrophage inflammatory protein-1β (CCL4) |
| BAFF | B-cell activating factor | IL-23 | Interleukin-23 | MIP-3α | Macrophage inflammatory protein-3α (CCL20) |
| CD30 | CD30 | IL-27 | Interleukin-27 | MMP-1 | Matrix metalloproteinase-1 |
| CD163 | CD163 | IL-2R | Interleukin-2R | NGF- β | Nerve Growth Factor-β |
| ENA-78 | Epithelial neutrophil-activating peptide 78  (CXCL5) | IL-4 | Interleukin-4 | S100B | S100B |
| Eotaxin | CCL11 | IL-5 | Interleukin-5 | TNF-α | Tumor necrosis factor- α |
| Eotaxin-3 | CCL26 | IL-6 | Interleukin-6 | TNF- β | Tumor necrosis factor- β |
| FGF-2 | Fibroblast Growth Factor-2 | IL-8 | Interleukin-8 | TNF-R2 | Tumor necrosis factor- receptor 2 |
| G-CSF | Granulocyte-colony stimulating factor | IL-9 | Interleukin-9 | TRAIL | Tumor necrosis factor (TNF)-related apoptosis-inducing ligand |
| GRO-α | Growth-related oncogene α (CXCL1) | IP-10 | Interferon-γ inducible protein-10 (CXCL10) | TWEAK | Tumor necrosis factor-like weak inducer of apoptosis |
| HGF | Hepatocyte Growth Factor | LIF | Leukemia inhibitory factor | VEGF-A | Vascular endothelial growth factor-A |
| IFN-α | Interferon-α | M-CSF | Macrophage colony-stimulating factor |  |  |
| IL-10 | Interleukin-10 | MCP-1 | Monocyte chemoattractant protein-1 (CCL2) |  |  |
| IL-15 | Interleukin-15 | MCP-2 | Monocyte chemoattractant protein-2 (CCL8) |  |  |
| IL-16 | Interleukin-16 | MCP-3 | Monocyte chemoattractant protein-3 (CCL7) |  |  |
| IL-18 | Interleukin-18 | MIF | Macrophage migration inhibitory factor |  |  |
| IL-1α | Interleukin-1α | MIG | Monokine induced by interferon gamma  (CXCL9) |  |  |
| IL-1β | Interleukin-1β | MIP-1α | Macrophage inflammatory protein-1α (CCL3) |  |  |

Table S2. Detectability of secreted factors in tumor tissue-conditioned media

| **Secreted factors** | **Samples between LLOQ and ULOQ** | **Secreted factors** | **Samples between LLOQ and ULOQ** | **Secreted factors** | **Samples between LLOQ and ULOQ** |
| --- | --- | --- | --- | --- | --- |
| **APRIL** | **55/55 (100%)^a^** | **IL-18** | **55/55 (100%)^a^** | **M-CSF** | **55/55 (100%)^a^** |
| **BAFF** | **53/55 (96%)^a^** | **IL-1α** | **55/55 (100%)^a^** | MDC | 31/55 (56%)^a^ |
| BLC | 22/55 (40%)^a^ | **IL-1β** | **54/55 (98%)^a^** | **MIF** | **55/55 (100%)^a^** |
| **CD30** | **55/55 (100%)^a^** | IL-2 | 23/55 (42%)^a^ | **MIG** | **43/55 (78%)^a^** |
| CD40L | 26/55 (47%)^a^ | IL-20 | 13/55 (24%)^a^ | **MIP-1α** | **55/55 (100%)^a^** |
| **CD163** | **67/67 (100%)^b^** | **IL-21** | **53/55 (96%)^a^** | **MIP-1β** | **48/55 (87%)^a^** |
| **ENA-78** | **47/55 (85%)^a^** | IL-22 | 31/55 (56%)^a^ | **MIP-3α** | **55/55 (100%)^a^** |
| **Eotaxin** | **51/55 (93%)^a^** | **IL-23** | **45/55 (82%)^a^** | **MMP1** | **55/55 (100%)^a^** |
| Eotaxin-2 | 20/55 (36%)^a^ | **IL-27** | **51/55 (93%)^a^** | **NGF- β** | **51/55 (93%)^a^** |
| **Eotaxin-3** | **55/55 (100%)^a^** | **IL-2R** | **52/55 (95%)^a^** | **S100 B** | **54/54 (100%)^a^** |
| **FGF-2** | **55/55 (100%)^a^** | IL-3 | 19/55 (35%)^a^ | SCF | 32/55 (58%)^a^ |
| Fractalkine | 31/55 (56%)^a^ | IL-31 | 11/55 (20%)^a^ | SDF-1α | 19/55 (35%)^a^ |
| **G-CSF** | **42/55 (76%)^a^** | **IL-4** | **55/55 (100%)^a^** | **TNF- β** | **36/55 (65%)^a^** |
| GM-CSF | 33/55 (60%)^a^ | **IL-5** | **55/55 (100%)^a^** | **TNF-R2** | **55/55 (100%)^a^** |
| **GRO-α** | **53/55 (96%)^a^** | **IL-6** | **55/55 (100%)^a^** | **TNF-α** | **62/91 (68%)^a^** |
| **HGF** | **55/55 (100%)^a^** | IL-7 | 20/55 (36%)^a^ | **TRAIL** | **55/55 (100%)^a^** |
| IFN-γ | 18/55 (33%)^a^ | **IL-8** | **55/55 (100%)^a^** | TSLP | 18/55 (33%)^a^ |
| **IFN-α** | **35/55 (64%)^a^** | **IL-9** | **55/55 (100%)^a^** | **TWEAK** | **55/55 (100%)^a^** |
| **IL-10** | **44/55 (80%)^a^** | **IP-10** | **54/55 (98%)^a^** | **VEGF-A** | **55/55 (100%)^a^** |
| IL-12p70 | 4/55 (7%)^a^ | I-TAC | 3/55 (5%)^a^ |  |  |
| IL-13 | 20/55 (36%)^a^ | **LIF** | **55/55 (100%)^a^** | |  |
| **IL-15** | **55/55 (100%)^a^** | **MCP-1** | **55/55 (100%)^a^** | |  |
| **IL-16** | **55/55 (100%)^a^** | **MCP-2** | **50/55 (91%)^a^** | |  |
| IL-17A | 29/55 (53%)^a^ | **MCP-3** | **48/55 (87%)^a^** | |  |

Secreted factors with an absolute concentration between the LLOQ and ULOQ calculated in more than 60% of tested samples were included in the study. Forty-seven out of 67 tested candidate biomarkers fulfilled the inclusion criteria (bold). Factors exceeding ULOQ were approximated with the corresponding ULOQ. Abbreviations: LLOQ, lower limit of quantification; ULOQ, upper limit of quantification. Notes: ^a^ Assessed with Luminex assay; ^b^ Assessed with ELISA assay.

Table S3. Descriptive statistics and distribution characteristics before outlier removal: assessment of data quality

| **Secreted factors** | **No.** | **Min. (pg/mL)** | **Med. (pg/mL)** | **Max. (pg/mL)** | **Mean (pg/mL)** | **Std. dev (pg/mL)** | **CV** | **Skewness** | **Kurtosis** | **No.Outliers** |
| --- | --- | --- | --- | --- | --- | --- | --- | --- | --- | --- |
|  | **Samples** |  |  |  |  |  |  |  |  | **(Q=0.1%)** |
|  |  |  |  |  |  |  |  |  |  |  |
| TNF-α | 91 | 0.00 | 4.33 | 1681.00 | 42.27 | 187.80 | 444.3% | 7.84 | 66.61 | 11 |
| APRIL | 55 | 136.20 | 3218.00 | 74905.00 | 7704.00 | 12568.00 | 163.1% | 3.54 | 15.34 | 7 |
| BAFF | 55 | 3.54 | 38.87 | 259.80 | 43.36 | 42.53 | 98.08% | 3.14 | 13.10 | 2 |
| CD30 | 55 | 11.92 | 82.78 | 213.50 | 90.38 | 47.35 | 52.39% | 0.67 | 0.10 | 0 |
| ENA-78 | 55 | 0.00 | 96.64 | 37500.00 | 2269.00 | 6550.00 | 288.7% | 4.29 | 19.64 | 12 |
| Eotaxin-3 | 55 | 6.55 | 21.30 | 49.14 | 21.24 | 7.26 | 34.18% | 1.34 | 4.44 | 1 |
| FGF-2 | 55 | 697.50 | 6816.00 | 46500.00 | 11353.00 | 13298.00 | 117.1% | 2.01 | 3.00 | 6 |
| IL-16 | 55 | 151.00 | 1109.00 | 4556.00 | 1280.00 | 921.10 | 71.96% | 1.98 | 4.61 | 3 |
| IL-2R | 55 | 0.00 | 470.10 | 13215.00 | 1091.00 | 1951.00 | 178.9% | 4.78 | 28.07 | 6 |
| MCP-2 | 55 | 0.27 | 1.52 | 15.29 | 2.51 | 2.78 | 111.1% | 2.90 | 9.45 | 5 |
| MCP-3 | 55 | 1.95 | 34.34 | 128.40 | 30.08 | 25.10 | 83.44% | 1.76 | 5.43 | 0 |
| MIF | 55 | 467.40 | 3228.00 | 9815.00 | 3227.00 | 2345.00 | 72.68% | 0.63 | -0.32 | 0 |
| MIG | 55 | 0.00 | 23.41 | 340.60 | 60.02 | 81.59 | 135.9% | 2.26 | 5.28 | 3 |
| TNF-R2 | 55 | 11.13 | 109.40 | 573.90 | 145.10 | 115.70 | 79.73% | 2.23 | 5.60 | 3 |
| TRAIL | 55 | 91.93 | 320.20 | 1715.00 | 358.40 | 293.90 | 82.01% | 2.62 | 8.54 | 4 |
| TWEAK | 55 | 3106.00 | 39758.00 | 168365.00 | 46391.00 | 33008.00 | 71.15% | 1.26 | 2.22 | 0 |
| NGF-β | 55 | 0.00 | 27.96 | 585.50 | 55.04 | 93.55 | 170.0% | 4.09 | 19.91 | 8 |
| Eotaxin | 55 | 0.80 | 4.10 | 16.56 | 4.78 | 3.26 | 68.12% | 1.98 | 4.33 | 3 |
| G-CSF | 55 | 0.46 | 24.78 | 4872.00 | 238.20 | 714.40 | 299.9% | 5.48 | 33.93 | 7 |
| GRO-α | 55 | 1.87 | 27.53 | 2168.00 | 243.50 | 483.70 | 198.6% | 2.57 | 6.17 | 10 |
| HGF | 55 | 445.60 | 2880.00 | 25595.00 | 4504.00 | 4897.00 | 108.7% | 2.72 | 8.64 | 4 |
| IFN-α | 55 | 1.23 | 5.55 | 43.82 | 9.83 | 10.33 | 105.1% | 2.30 | 4.25 | 6 |
| IL-1α | 55 | 1.75 | 23.90 | 280.50 | 45.53 | 55.31 | 121.5% | 2.59 | 7.77 | 3 |
| IL-1β | 55 | 5.68 | 28.69 | 667.90 | 55.96 | 101.20 | 180.8% | 4.68 | 25.74 | 6 |
| IL-10 | 55 | 0.26 | 22.64 | 99.84 | 29.16 | 28.58 | 97.99% | 0.84 | -0.32 | 0 |

Table S3 (continued). Descriptive statistics and distribution characteristics before outlier removal: assessment of data quality

| **Secreted factors** | **No.** | **Min. (pg/mL)** | **Med. (pg/mL)** | **Max. (pg/mL)** | **Mean (pg/mL)** | **Std. dev (pg/mL)** | **CV** | **Skewness** | **Kurtosis** | **No.Outliers** |
| --- | --- | --- | --- | --- | --- | --- | --- | --- | --- | --- |
|  | **Samples** |  |  |  |  |  |  |  |  | **(Q=0.1%)** |
|  |  |  |  |  |  |  |  |  |  |  |
| IL-15 | 55 | 5.62 | 37.41 | 115.50 | 43.70 | 29.83 | 68.27% | 0.86 | -0.09 | 0 |
| IL-18 | 55 | 7.64 | 91.33 | 1665.00 | 175.90 | 252.10 | 143.4% | 4.49 | 23.80 | 3 |
| IL-21 | 55 | 4.12 | 15.02 | 507.70 | 74.09 | 102.50 | 138.4% | 2.51 | 7.49 | 2 |
| IL-23 | 55 | 0.00 | 45.53 | 315.30 | 79.74 | 84.78 | 106.3% | 1.19 | 0.67 | 0 |
| IL-27 | 55 | 6.36 | 159.70 | 1038.00 | 264.30 | 265.30 | 100.4% | 1.17 | 0.79 | 2 |
| IL-4 | 55 | 43.78 | 196.70 | 653.30 | 236.30 | 157.50 | 66.66% | 1.05 | 0.43 | 0 |
| IL-5 | 55 | 8.00 | 50.27 | 175.00 | 49.73 | 33.82 | 68.01% | 1.19 | 2.45 | 0 |
| IL-6 | 55 | 71.06 | 3291.00 | 40546.00 | 5161.00 | 7099.00 | 137.5% | 3.04 | 11.80 | 9 |
| IL-8 | 55 | 1329.00 | 16447.00 | 81500.00 | 34856.00 | 32653.00 | 93.68% | 0.60 | -1.49 | 0 |
| IL-9 | 55 | 49.71 | 343.30 | 1004.00 | 441.10 | 244.50 | 55.42% | 0.60 | -0.70 | 0 |
| IP-10 | 55 | 2.45 | 52.66 | 349.70 | 72.39 | 71.65 | 98.98% | 1.64 | 3.34 | 1 |
| LIF | 55 | 168.70 | 1373.00 | 6885.00 | 2106.00 | 1771.00 | 84.07% | 1.24 | 0.70 | 2 |
| MCP-1 | 55 | 506.60 | 9859.00 | 37750.00 | 10743.00 | 9546.00 | 88.86% | 1.75 | 2.83 | 4 |
| M-CSF | 55 | 371.60 | 4482.00 | 13682.00 | 5147.00 | 3825.00 | 74.32% | 0.63 | -0.57 | 0 |
| MIP-1α | 55 | 8.32 | 44.37 | 513.90 | 67.09 | 79.42 | 118.4% | 3.74 | 18.31 | 6 |
| MIP-1β | 55 | 0.00 | 44.91 | 678.20 | 99.79 | 136.00 | 136.3% | 2.45 | 6.54 | 5 |
| MIP-3α | 55 | 11.82 | 263.00 | 15871.00 | 1286.00 | 2404.00 | 186.9% | 4.41 | 25.17 | 1 |
| MMP-1 | 55 | 8.01 | 381.20 | 17200.00 | 2125.00 | 4141.00 | 194.8% | 2.52 | 5.73 | 9 |
| TNF-β | 55 | 0.00 | 6.53 | 140.60 | 20.34 | 31.35 | 154.1% | 2.76 | 7.61 | 9 |
| VEGF-A | 55 | 3865.00 | 31605.00 | 215000.00 | 76737.00 | 81629.00 | 106.4% | 0.99 | -0.77 | 13 |
| CD163 | 67 | 40.00 | 3646.00 | 53016.00 | 6602.00 | 9565.00 | 144.9% | 3.10 | 10.98 | 5 |
| S100B | 54 | 79.72 | 180.90 | 1818.00 | 226.20 | 242.70 | 107.3% | 5.65 | 36.25 | 2 |

The Robust Regression and Outlier Removal (ROUT) method in GraphPad Prism software was employed to identify outliers. The maximum acceptable false discovery rate (Q parameter) was set to 0.1%, enabling the detection of the most extreme outliers while minimizing the risk of false positives. Abbreviations: No., number; Min., minimum value in dataset; Med., median; Max., maximum value in dataset; Mean, mean value in dataset; Std. dev., Standard deviation; CV., coefficient of variation.

Table S4. Descriptive statistics and distribution characteristics after outlier removal: impact on data quality and distribution

| **Secreted factors** | **No.** | **Min. (pg/mL)** | **Med. (pg/mL)** | **Max. (pg/mL)** | **Mean (pg/mL)** | **Std. dev (pg/mL)** | **CV** | **Skewness** | **Kurtosis** | **No.Outliers** |
| --- | --- | --- | --- | --- | --- | --- | --- | --- | --- | --- |
|  | **Samples** |  |  |  |  |  |  |  |  | **(Q=0.1%)** |
|  |  |  |  |  |  |  |  |  |  |  |
| TNF-α | 80 | 0.00 | 3.53 | 31.54 | 6.59 | 7.90 | 119.9% | 1.42 | 1.30 | 0 |
| APRIL | 48 | 136.20 | 2426.00 | 13542.00 | 3831.00 | 3437.00 | 89.70% | 1.23 | 0.53 | 0 |
| BAFF | 53 | 3.54 | 38.61 | 121.20 | 36.78 | 24.40 | 66.35% | 0.99 | 1.71 | 0 |
| CD30 | 55 | 11.92 | 82.78 | 213.50 | 90.38 | 47.35 | 52.39% | 0.67 | 0.10 | 0 |
| ENA-78 | 43 | 0.00 | 57.14 | 871.60 | 159.70 | 216.70 | 135.7% | 1.50 | 1.60 | 0 |
| Eotaxin-3 | 54 | 6.55 | 21.25 | 44.16 | 20.72 | 6.22 | 30.03% | 0.66 | 2.85 | 0 |
| FGF-2 | 49 | 697.50 | 5790.00 | 21014.00 | 7049.00 | 5062.00 | 71.81% | 0.81 | 0.23 | 0 |
| IL-16 | 52 | 151.00 | 1063.00 | 2658.00 | 1106.00 | 573.80 | 51.89% | 0.65 | 0.27 | 0 |
| IL-2R | 49 | 0.00 | 422.30 | 2316.00 | 604.40 | 595.40 | 98.51% | 1.41 | 1.40 | 0 |
| MCP-2 | 50 | 0.27 | 1.35 | 4.77 | 1.76 | 1.11 | 62.73% | 1.26 | 0.76 | 0 |
| MCP-3 | 55 | 1.95 | 34.34 | 128.40 | 30.08 | 25.10 | 83.44% | 1.76 | 5.43 | 0 |
| MIF | 55 | 467.40 | 3228.00 | 9815.00 | 3227.00 | 2345.00 | 72.68% | 0.63 | -0.32 | 0 |
| MIG | 52 | 0.00 | 23.04 | 180.00 | 44.09 | 48.00 | 108.9% | 1.20 | 0.41 | 0 |
| TNF-R2 | 52 | 11.13 | 106.60 | 332.20 | 123.60 | 72.00 | 58.25% | 1.24 | 1.16 | 0 |
| TRAIL | 51 | 91.93 | 273.80 | 792.90 | 290.70 | 149.10 | 51.31% | 0.97 | 1.31 | 0 |
| TWEAK | 55 | 3106.00 | 39758.00 | 168365.00 | 46391.00 | 33008.00 | 71.15% | 1.26 | 2.22 | 0 |
| NGF-β | 47 | 0.00 | 26.26 | 99.43 | 25.93 | 18.98 | 73.19% | 1.44 | 3.81 | 0 |
| Eotaxin | 52 | 0.80 | 3.97 | 10.85 | 4.19 | 2.14 | 51.10% | 1.20 | 2.24 | 0 |
| G-CSF | 48 | 0.46 | 17.91 | 247.40 | 53.22 | 66.27 | 124.5% | 1.64 | 1.97 | 0 |
| GRO-α | 45 | 1.87 | 17.06 | 326.10 | 50.20 | 73.58 | 146.6% | 2.12 | 4.49 | 0 |
| HGF | 51 | 445.60 | 2566.00 | 9804.00 | 3381.00 | 2406.00 | 71.17% | 1.04 | 0.17 | 0 |
| IFN-α | 49 | 1.23 | 5.35 | 20.49 | 6.46 | 3.31 | 51.27% | 1.67 | 5.33 | 0 |
| IL-1α | 52 | 1.75 | 23.30 | 104.20 | 34.41 | 29.40 | 85.44% | 0.77 | -0.74 | 0 |
| IL-1β | 49 | 5.68 | 19.57 | 105.40 | 30.18 | 24.84 | 82.32% | 1.51 | 1.87 | 0 |
| IL-10 | 55 | 0.26 | 22.64 | 99.84 | 29.16 | 28.58 | 97.99% | 0.84 | -0.32 | 0 |

Table S4 (continued). Descriptive statistics and distribution characteristics after outlier removal: impact on data quality and distribution

| **Secreted factors** | **No.** | **Min. (pg/mL)** | **Med. (pg/mL)** | **Max. (pg/mL)** | **Mean (pg/mL)** | **Std. dev (pg/mL)** | **CV** | **Skewness** | **Kurtosis** | **No.Outliers** |
| --- | --- | --- | --- | --- | --- | --- | --- | --- | --- | --- |
|  | **Samples** |  |  |  |  |  |  |  |  | **(Q=0.1%)** |
|  |  |  |  |  |  |  |  |  |  |  |
| IL-15 | 55 | 5.62 | 37.41 | 115.50 | 43.70 | 29.83 | 68.27% | 0.86 | -0.09 | 0 |
| IL-18 | 52 | 7.64 | 89.75 | 355.80 | 127.70 | 93.78 | 73.43% | 1.25 | 0.47 | 0 |
| IL-21 | 53 | 4.12 | 14.60 | 311.80 | 59.05 | 67.31 | 114.0% | 1.46 | 2.24 | 0 |
| IL-23 | 55 | 0.00 | 45.53 | 315.30 | 79.74 | 84.78 | 106.3% | 1.19 | 0.67 | 0 |
| IL-27 | 53 | 6.36 | 158.40 | 878.10 | 236.90 | 227.70 | 96.14% | 0.98 | 0.23 | 0 |
| IL-4 | 55 | 43.78 | 196.70 | 653.30 | 236.30 | 157.50 | 66.66% | 1.05 | 0.43 | 0 |
| IL-5 | 55 | 8.00 | 50.27 | 175.00 | 49.73 | 33.82 | 68.01% | 1.19 | 2.45 | 0 |
| IL-6 | 46 | 71.06 | 2365.00 | 9197.00 | 2672.00 | 2298.00 | 86.01% | 1.06 | 0.87 | 0 |
| IL-8 | 55 | 1329.00 | 16447.00 | 81500.00 | 34856.00 | 32653.00 | 93.68% | 0.60 | -1.49 | 0 |
| IL-9 | 55 | 49.71 | 343.30 | 1004.00 | 441.10 | 244.50 | 55.42% | 0.60 | -0.70 | 0 |
| IP-10 | 54 | 2.45 | 52.00 | 249.90 | 67.25 | 61.26 | 91.09% | 1.12 | 0.75 | 0 |
| LIF | 53 | 168.70 | 1323.00 | 6187.00 | 1927.00 | 1536.00 | 79.70% | 1.16 | 0.54 | 0 |
| MCP-1 | 51 | 506.60 | 9569.00 | 28569.00 | 8625.00 | 5958.00 | 69.08% | 1.18 | 2.82 | 0 |
| M-CSF | 55 | 371.60 | 4482.00 | 13682.00 | 5147.00 | 3825.00 | 74.32% | 0.63 | -0.57 | 0 |
| MIP-1α | 49 | 8.32 | 39.88 | 138.20 | 45.36 | 28.90 | 63.70% | 1.20 | 1.47 | 0 |
| MIP-1β | 50 | 0.00 | 35.17 | 260.10 | 63.05 | 61.99 | 98.33% | 1.24 | 1.03 | 0 |
| MIP-3α | 54 | 11.82 | 258.10 | 5043.00 | 1016.00 | 1341.00 | 132.0% | 1.33 | 0.84 | 0 |
| MMP-1 | 46 | 8.01 | 314.10 | 1946.00 | 502.90 | 565.10 | 112.4% | 1.47 | 1.03 | 0 |
| TNF-β | 46 | 0.00 | 6.37 | 33.74 | 9.16 | 8.38 | 91.43% | 1.78 | 2.32 | 0 |
| VEGF-A | 42 | 3865.00 | 27400.00 | 116012.00 | 33941.00 | 28956.00 | 85.31% | 1.39 | 1.48 | 0 |
| CD163 | 62 | 40.00 | 2822.00 | 15718.00 | 4334.00 | 4055.00 | 93.55% | 1.14 | 0.54 | 0 |
| S100B | 52 | 79.72 | 179.30 | 391.80 | 186.50 | 74.72 | 40.06% | 0.93 | 0.77 | 0 |

The Robust Regression and Outlier Removal (ROUT) method in GraphPad Prism software was employed to identify and exclude outliers. The maximum acceptable false discovery rate (Q parameter) was set to 0.1%, enabling the detection of the most extreme outliers while minimizing the risk of false positives. Abbreviations: No., number; Min., minimum value in dataset; Med., median; Max., maximum value in dataset; Mean, mean value in dataset; Std. dev., Standard deviation; CV., coefficient of variation.
